# Movement ecology of vulnerable lowland tapirs between areas of varying human disturbance

**DOI:** 10.1186/s40462-022-00313-w

**Published:** 2022-03-14

**Authors:** E. P. Medici, S. Mezzini, C. H. Fleming, J. M. Calabrese, M. J. Noonan

**Affiliations:** 1 Lowland Tapir Conservation Initiative (LTCI), Instituto de Pesquisas Ecológicas (IPÊ), Rodovia Dom Pedro I, km 47, Nazaré Paulista, São Paulo 12960-000 Brazil; 2 IUCN SSC Tapir Specialist Group (TSG), Campo Grande, Brazil; 3 Escola Superior de Conservação Ambiental E Sustentabilidade (ESCAS/IPÊ), Rodovia Dom Pedro I, km 47, Nazaré Paulista, São Paulo 12960-000 Brazil; 4 The Irving K. Barber Faculty of Science, The University of British Columbia, Okanagan Campus, Kelowna, Canada; 5 University of Maryland College Park, College Park, MD USA; 6 Smithsonian Conservation Biology Institute, Front Royal, VA USA; 7 Center for Advanced Systems Understanding (CASUS), Görlitz, Germany; 8 Helmholtz-Zentrum Dresden Rossendorf (HZDR), Dresden, Germany; 9 Department of Ecological Modelling, Helmholtz Centre for Environmental Research (UFZ), Leipzig, Germany

**Keywords:** Anthropocene, Continuous-time movement modelling, Home range, Human Footprint Index, Space use

## Abstract

**Background:**

Animal movement is a key ecological process that is tightly coupled to local environmental conditions. While agriculture, urbanisation, and transportation infrastructure are critical to human socio-economic improvement, these have spurred substantial changes in animal movement across the globe with potential impacts on fitness and survival. Notably, however, human disturbance can have differential effects across species, and responses to human activities are thus largely taxa and context specific. As human disturbance is only expected to worsen over the next decade it is critical to better understand how species respond to human disturbance in order to develop effective, case-specific conservation strategies.

**Methods:**

Here, we use an extensive telemetry dataset collected over 22 years to fill a critical knowledge gap in the movement ecology of lowland tapirs (*Tapirus terrestris*) across areas of varying human disturbance within three biomes in southern Brazil: the Pantanal, Cerrado, and Atlantic Forest.

**Results:**

From these data we found that the mean home range size across all monitored tapirs was 8.31 km^2^ (95% CI 6.53–10.42), with no evidence that home range sizes differed between sexes nor age groups. Interestingly, although the Atlantic Forest, Cerrado, and Pantanal vary substantially in habitat composition, levels of human disturbance, and tapir population densities, we found that lowland tapir movement behaviour and space use were consistent across all three biomes. Human disturbance also had no detectable effect on lowland tapir movement. Lowland tapirs living in the most altered habitats we monitored exhibited movement behaviour that was comparable to that of tapirs living in a near pristine environment.

**Conclusions:**

Contrary to our expectations, although we observed individual variability in lowland tapir space use and movement, human impacts on the landscape also had no measurable effect on their movement. Lowland tapir movement behaviour thus appears to exhibit very little phenotypic plasticity in response to human disturbance. Crucially, the lack of any detectable response to anthropogenic disturbance suggests that human modified habitats risk being ecological traps for tapirs and this information should be factored into conservation actions and species management aimed towards protecting lowland tapir populations.

**Supplementary Information:**

The online version contains supplementary material available at 10.1186/s40462-022-00313-w.

## Background

While agriculture, urbanisation, and transportation infrastructure are critical to human socio-economic improvement [17], the associated habitat transformations represent a major threat to species survival [18, 54, 76]. Of particular concern is the impact of human activities on animal movement and space use [3, 16, 71]. Animal movement governs how individuals, populations, and species interact with each other and the environment [29, 39, 63] and mediates key ecological processes [6]. The capacity for individuals to move unhindered across complex landscapes is therefore critical for species survival and ecosystem function. Problematically, human development has reduced the amount of habitat available to wildlife [8, 13, 32]. This has spurred substantial changes in animal movement behaviour across the globe [16, 19, 71], with potential consequences including reduced fitness and survival, altered predator–prey dynamics, reduced seed dispersal, genetic isolation and local extinction [14, 15, 19, 72].

Notably, human disturbance has been shown to have differential effects across species [16, 70], even for closely related taxa occupying the same habitat [68]. Responses to human activities are thus largely context specific [16] and cannot be expected to be consistent across taxa. For instance, while Wall et al. [78] found a tendency for African elephants (*Loxodonta spp.*) to exhibit reduced movement in human modified landscapes, Morato et al. [47] noted that jaguars (*Panthera onca*) living in regions with high human population densities in South America occupied home ranges that were orders of magnitude larger than those of jaguars living in more pristine habitats. As human disturbance is only expected to worsen over the next decade it is critical to better understand how species respond to human disturbance to develop effective, case-specific conservation strategies.

Here we focus on understanding how the movement behaviour of lowland tapirs (*Tapirus terrestris*) varies across areas of varying human disturbance within the Pantanal, Cerrado, and Atlantic Forest biomes in southern Brazil. Lowland tapirs are herbivores of the order Perissodactyla that can reach over 2.5 m in length and weigh up to 250 kg [40]. While lowland tapirs are distributed throughout South America [27], their populations have suffered severe reductions, with local and regional extirpations, and are currently classified as vulnerable to extinction [74]. Although the incorporation of information on animal movement is a key component in designing effective conservation and recovery strategies [3], currently, very little is known about the movement ecology of tapirs (but see [22, 52, 69]). This knowledge gap is especially pertinent given that large terrestrial mammals, such as tapirs, tend to have larger home ranges and greater absolute mobility than do small mammals [11, 51], making them more susceptible to anthropogenic impacts than smaller bodied species [31, 71]. Here, we use an extensive telemetry dataset collected over 22 years to describe the movement ecology of tapirs and study how changes in habitat composition and human disturbance influence their movement and space use. First, animals living in highly productive environments do not need to range over wide areas to meet their energetic needs [35, 48, 57]. As such, we expected that tapirs should exhibit plasticity in their movement and space use in relation to local environmental conditions as well as biome type. Furthermore, because human activity tends to result in increased movement for large herbivores [16] our underlying hypothesis was that tapirs should exhibit greater movement distances and larger home-range areas when living in human-modified landscapes.

## Methods

### Study area and data collection

**Fig. 1:**
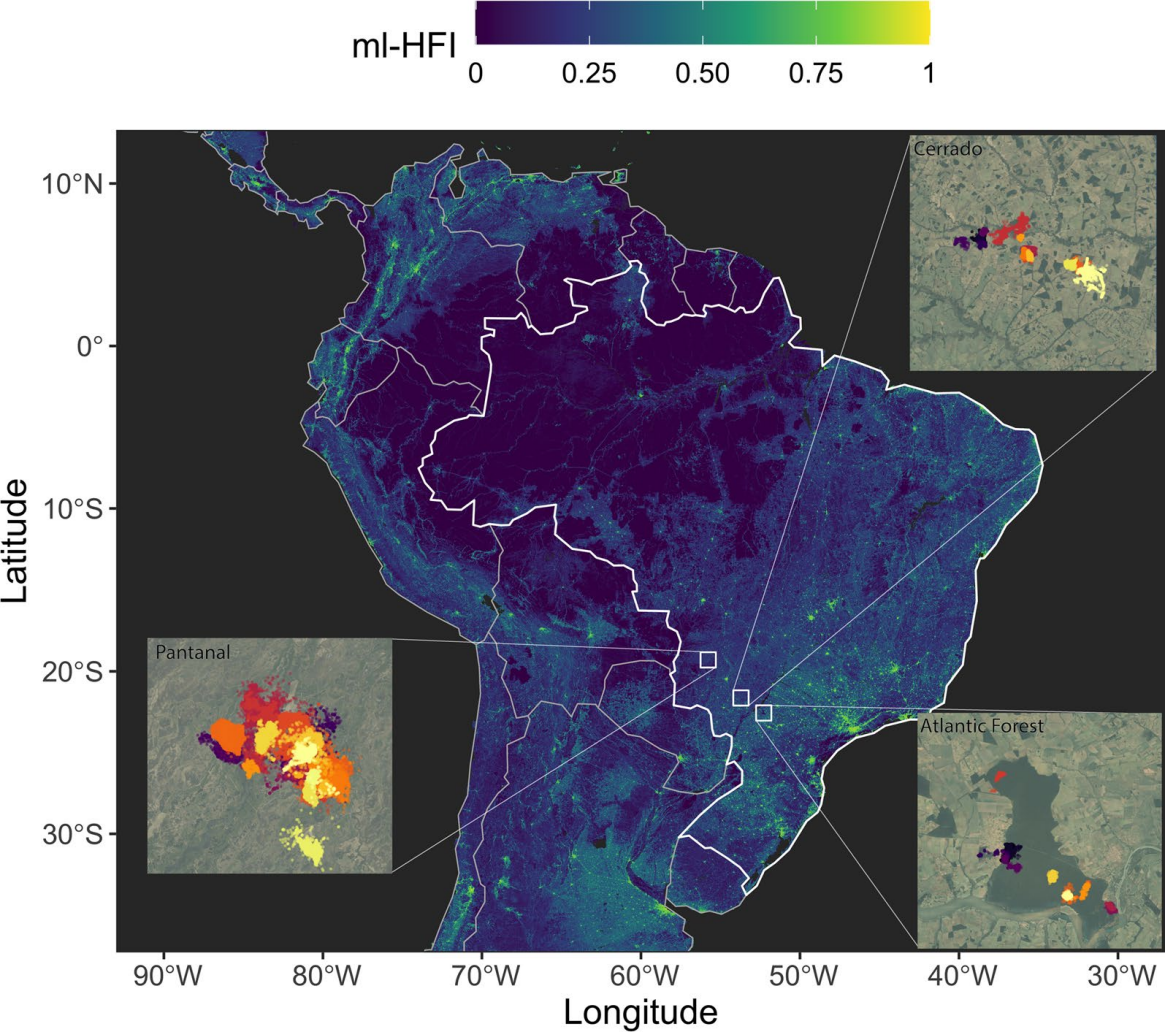
Location of the three study sites (Pantanal, Cerrado, Atlantic Forest) over a raster of machine-learning-based human footprint index (ml-HFI), an index of human pressure on the landscape that is derived from remotely sensed surface imagery and ranges on a scale between 0 (no human impact), and 1 (high human impact). The Atlantic Forest was the most disturbed biome we monitored tapirs in with only ca. 12–29% of the natural habitat remaining, whereas the Cerrado has lost almost 50% of its natural area, and the Pantanal is a nearly pristine biome

The data were collected in three different biomes in southern Brazil (Fig. 1): Atlantic Forest (1997–2007), Pantanal (2008–2019), and south-western Cerrado (2016–2018).

### Atlantic forest

Morro do Diabo State Park is a protected area located in the Municipality of Teodoro Sampaio (22°32’S, 52°18’W), state of São Paulo, in the southeastern region of Brazil. The park has an area of 370 km^2^ composed of a mosaic of mature and secondary deciduous forest, surrounded by the Paranapanema River in the south, and of a matrix of cattle ranches and agriculture, mostly sugar cane, in the remaining borders [73]. Its average annual temperature is 22 °C and annual rainfall is 1347 mm [28]. The park is part of the “Planalto Forest,” which is distinguished from the coastal forest of the Atlantic Forest biome by having lower annual rainfall and a marked dry season from May to September and is thus more similar to the Cerrado biome [61]. In fact, the semi-deciduous forests of the “Planalto Forest” are similar to those occurring within or on the edges of the Cerrado [61]. The Atlantic Forest was the most disturbed biome we monitored tapirs in, with only ca. 12–29% of the natural habitat remaining [37, 58, 59, 66].

### Cerrado

The study site in the Cerrado biome is a 2200 km^2^ mosaic of private properties (cattle ranches and farms) and landless people settlements within the Municipalities of Nova Alvorada do Sul and Nova Andradina, Mato Grosso do Sul State (21°37’S, 53°40’W). The area includes small fragments of natural Cerrado habitat (Cerradão fragments, gallery forests, and marshland—25% of the study area), surrounded by areas highly impacted by human activities such as agriculture (particularly sugarcane, soybean and corn), cattle-ranching (cultivated pastureland), eucalyptus plantations, rural communities, and highways. The average annual temperature is 25 °C and annual rainfall is 1185 mm. The Cerrado has lost almost 50% of its natural area due primarily to human driven land-use change to agriculture and cattle ranching [36, 45].

### Pantanal

Baía das Pedras Ranch, a private property of 145 km^2^, is located in the Nhecolândia Sub-Region of the Southern Pantanal, Municipality of Aquidauana (19°20’S, 55°43’W), Mato Grosso do Sul State, in the central-western region of Brazil. The ranch includes a mosaic of seasonally inundated grasslands, lakes, gallery forests, scrub, and deciduous forests that supports an abundance of wildlife and is situated far away from the edges of the biome where deforestation and other anthropogenic threats are occurring. Cattle are raised extensively on the native grasses. The average annual temperature is 25 °C and annual rainfall is 1185 mm [12]. The Pantanal is a nearly pristine biome with substantially less human disturbance than the Atlantic Forest and Cerrado.

In each study site, tapirs were captured by darting after physical restraint in either box traps or pitfall traps, or by darting from a distance [55]. Animals were anesthetized mostly using a combination of butorphanol, medetomidine and ketamine, as described by Medici et al. [41] and Fernandes-Santos et al. [20]. Reversal agents were administrated at the end of procedures. The procedures carried out during immobilization included the subcutaneous insertion of a microchip, morphometric measurements, sex and age class determination, physical examination, collection of biological samples for health and genetic studies, and placement of a telemetry collar on adults. Animals were tracked using VHF tracking (all three regions,Telonics® MOD500) and GPS tracking (Pantanal and Cerrado; Telonics® TGW SOB and GPS IRIDIUM models). A total of 74 tapirs were tracked starting in July of 1997 until October of 2019, with the majority of the individuals being in the Pantanal (46), while 17 and 11 were from the Cerrado and Atlantic Forest regions, respectively.

Tapirs equipped with VHF collars were monitored for 5 days per month with data collection concentrated during crepuscular times, 3 h at dawn (04:00–07:00 h) and 3 h at dusk (17:00–20:00 h). These periods are the two main peaks of tapir activity [40]. Each tapir was located every 30 min during the sampling periods. GPS collars were programmed to obtain a fix every hour and operated for a median of 15.4 months across all tagged tapirs. GPS fix success rates were 75% in the Pantanal and 90% in the Cerrado. The full dataset comprised 232,622 location estimates collected over a period of 22 years (for full details see Additional file 1: File S1). In addition to the tapir location data, we collected 883 and 174 measurements from tags in fixed locations in the Pantanal and Cerrado, respectively in order to calibrate the measurement error of the GPS tracking collars.

### Data analysis

Initial exploratory analyses were carried out in ctmmweb (version 0.2.11, [10]. All formal statistical analysis and plotting were performed using R (version 4.0.5, R Core Team 2021 [56]), with the packages ctmm (version 0.6.1, [9], mgcv (version 1.8-36, [80], ggplot2 (version 3.3.4, [79], ggmap (version 3.0.0, [33]. The furrr package (version 0.2.2, [75] was used for parallel computation on Windows machines. All R code can be found in the GitHub repository at https://github.com/StefanoMezzini/tapirs. Details on the analyses are presented in Additional file 2: Appendix S2.

### Data calibration and cleaning

Before analysis, we performed an error calibration and data cleaning process to minimise the impacts of GPS measurement error and outliers on our subsequent analyses [21]. Data cleaning and calibration were carried out using the methods implemented in the ctmm R package. For this process, measurement error for location estimates collected via VHF telemetry was assumed to be insubstantial relative to the coarsely sampled movement data (median step length: 260.7 m) and raw locations were carried forward in the analyses. Measurement error on the GPS data was calibrated using a unitless Horizontal Dilution of Precision (HDOP), which quantifies the accuracy of each positional fix. We then estimated an equivalent range error with the HDOP values from the tags in fixed locations. This allowed for the unitless HDOP values to be converted into estimates of measurement error in meters. Notably, we found no effect of sampling technique, and thus error handling protocols, on home range area estimates (see Additional file 1: Appendix S1). After calibration, data points were considered as outliers (and removed) if they had a large (error-informed) distance from the median location and the minimum speed required to explain the displacement was unusually high (≥ $1$m/s). The Atlantic Forest dataset contained a total of 4,082 observations, 8 (ca. 0.2%) of which were removed as outliers; the Pantanal dataset contained 139,138 observations, 914 (ca. 0.7%) of which were removed; while the Cerrado dataset contained 90,402 observations, 193 (ca. 0.2%) of which were removed.

### Movement modelling and home range estimation

For each of the monitored tapirs we quantified a number of key movement metrics and home range-related characteristics that allowed us to test for an effect of habitat composition and human disturbance on tapir movement behaviour. For this we first identified the best Continuous-Time Movement Model (CTMM) for each animal using the ctmm.select function from the ctmm package. This fits a series of CTMMs to location data using perturbative Hybrid Residual Maximum Likelihood (pHREML, [22] and chooses the best model using small-sample-sized corrected Akaike’s Information Criterion (AICc). The models used here are insensitive to sampling frequency (Johnson et al. 2008; [7, 24]) and they account for spatio-temporal autocorrelation in the data (when necessary), so they are robust to irregular or frequent sampling frequency [23]. The parameter estimates from each individual’s movement model provided information on the tapir’s home range crossing time (${\tau }_{p}$, in days), and directional persistence timescale (${\tau }_{v}$, in hours).

We then conditioned on the selected CTMMs to estimate each animal’s 95% home range (HR) area (in km ${}^{2}$) using small-sample-size bias corrected Autocorrelated Kernel Density Estimation (AKDE, [25, 49], and average daily movement speed (in km/day) using continuous-time speed and distance (CTSD) estimation [50].

### Movement pattern analyses

We were first interested in understanding whether home-range areas and movement metrics differed across the three biomes, as well as between animals of different age and sex. For these comparisons, home-range estimates were compared using the meta-analysis methods implemented in the ctmm package, which treats the individual home-range area estimates as having a chi-squared sampling distribution, and the population of home-range areas as having an inverse-Gaussian distribution [26]. This approach also allowed for uncertainty in the individual home-range estimates to be propagated to the population-level estimates. Home-range crossing time, directional persistence, and mean movement speed were analysed using generalized linear models (GLMs) with a Gamma distribution and a log link function for the response. The GLMs were fit using the mgcv package [80] and Residual Maximum Likelihood (REML). Notably, because different tracking technologies were used to collect the tapir location data, we assessed whether tracking technique in-and-of-itself could have impacted the individual home range estimates. From these analyses found no effect of sampling technique on home range area (see Additional file 1: Appendix S1 for full details).

To test whether tapirs responded to different environment types, the HR sizes and average daily speeds were regressed against the proportions of the habitat types in each HR. For the Atlantic Forest, we used the habitat map provided in the park’s management plan [28]. For the Pantanal and Cerrado, we obtained satellite imagery from the periods of data collection. Habitat classification was then carried out using GIS software, and a team of researchers confirmed the classifications in the field. The primary habitat types included: (1) forest, (2) savannah, (3) exposed soil, (4) floodplain, and (5) water. For full details on the habitat composition of the different study areas see Additional file 1: Figure S2 in Appendix S1. Similarly, the HR sizes and average daily speeds were regressed against their HR’s average machine-learning-based human footprint index (ml-HFI) [34] to test whether human activity significantly altered the animals’ behavior. Briefly, convolutional neural networks, are used to identify patterns of human activity from the Hansen Global Forest Change imagery version 1.7 (GFCv1.7,for full details see [34]. The resulting ml-HFI is an index of human pressure on the landscape that is derived from remotely sensed surface imagery and ranges on a scale between 0 (no human impact), and 1 (high human impact). For these models we applied Generalized Additive Models (GAMs) with a Gamma distribution and a log link function for the response. The Gamma distribution allows for more accurate significance testing and is an appropriate distribution for variables that range between 0 and $\infty$, while the log link scale allows HFI to have a multiplicative effect on the response. The GAMs were fit using the mgcv package [80] and REML, and the best model was selected using AICc based model selection. All analyses were carried out at both the 95% and 50% quantiles. The findings were consistent between quantiles and only results at the 95% quantile are presented in the main text. Full results for the 50% core home ranges are presented in Additional file 2: Appendix S2.

## Results

### Individual variation in movement and space use

**Fig. 2:**
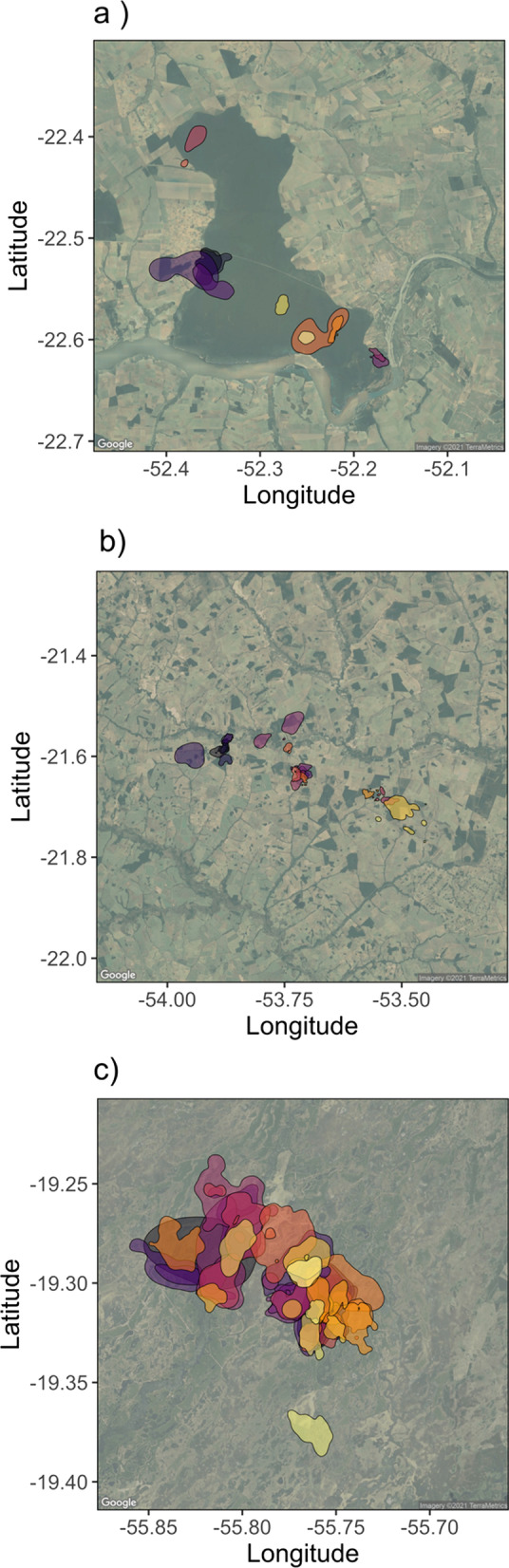
Autocorrelated kernel density estimates of each tapir’s 95% home range in each of the three regions: **a** Atlantic forest, **b** Cerrado, and **c** pantanal

**Fig. 3:**
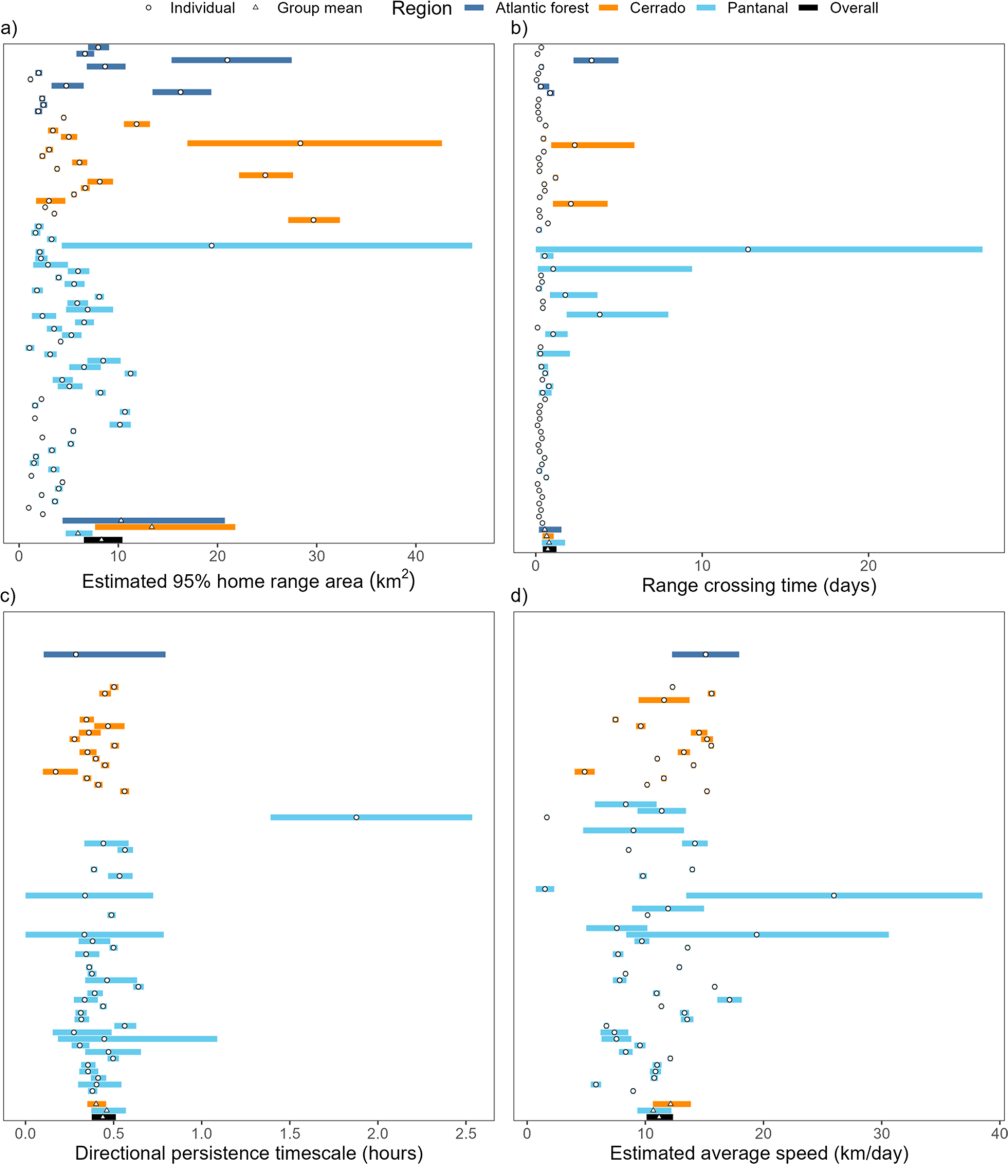
Parameter estimates from each tapir’s movement model (circles) and group means (triangles), with 95% confidence intervals. Individuals with a movement model that does not allow for inferences in movement speed are left blank

**Fig. 4:**
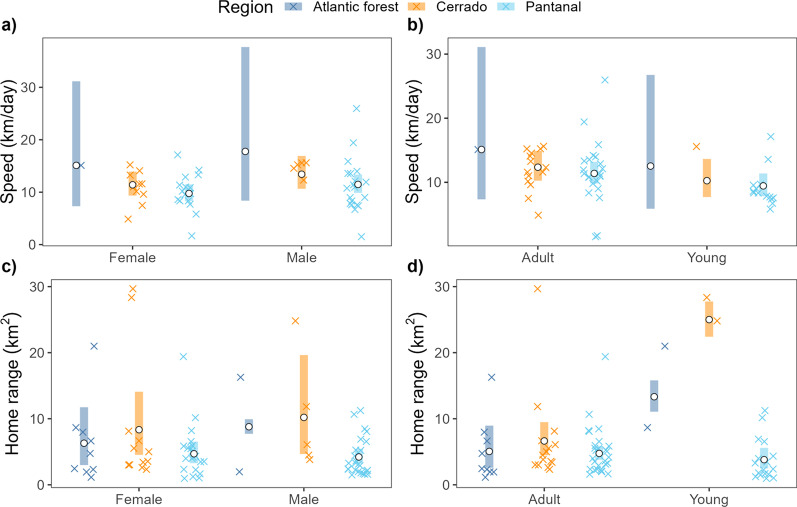
Daily average speed (**a**, **b**) and estimated home range size (**c**, **d**) by sex and age group across the three different biomes. White points and the shaded bands depict the population-level means and 95% confidence intervals. We note that estimation of movement speeds for adult females was only possible for a single tapir in the Atlantic Forest. In addition, we could only estimate speed for a single young tapir in the Cerrado

The mean home-range size across all monitored tapirs was 8.31 km^2^ (95% CI: 6.53—10.42; Fig. 2), ranging between 1.0 km^2^ and 29.7 km^2^ (Fig. 3a). Tapirs had HR crossing times of 0.72 days on average (95% CI 0.42–1.25), ranging from 0.05 to 12.8 days (Fig. 3b), and a mean velocity autocorrelation timescale of 0.44 h (95% CI 0.38–0.51), ranging from 0.17 to 1.88 h (Fig. 3c). We estimated that tapirs had mean movement speeds of 11.2 km/day (95% CI 10.1–12.3), ranging from 1.51 to 25.96 km/day (Fig. 3d). There was no evidence that average daily speed differed between sexes (females: 10.5 km/day, 95% CI 9.19–12.0; males: 11.9 km/day; 95% CI 10.3–13.7, $p=0.22$, 4a), nor between age groups (adults: 11.8 km/day, 95% CI 10.6–13.2; sub-adults: 9.5 km/day, 95% CI 7.9–11.4; $p=0.053$, Fig. 4b).

There was no evidence that home range sizes differed between sexes (males: 5.46 km^2^, 95% CI 4.03–7.23; females: 6.11 km^2^, 95% CI: 4.53–8.07 Fig. 4c), nor between age groups (adults: 5.37 km^2^, 95% CI: 4.39–11.64; sub-adults: 6.98 km^2^, 95% CI: 3.87–11.65; Fig. 4d). We estimated the male/female ratio of mean home-range areas to be 0.87 (0.56–1.30), and the adult/subadult ratio to be 0.70 (0.37–1.32), both of which include 1 and are thus non-significant.

### Variation in movement across biomes and variation in human disturbance

The Atlantic Forest, Cerrado, and Pantanal vary substantially in habitat composition, levels of human disturbance, and tapir population densities. Despite these differences, we found that lowland tapir movement behaviour and space use were consistent across all three biomes (Fig. 3).

**Fig. 5:**
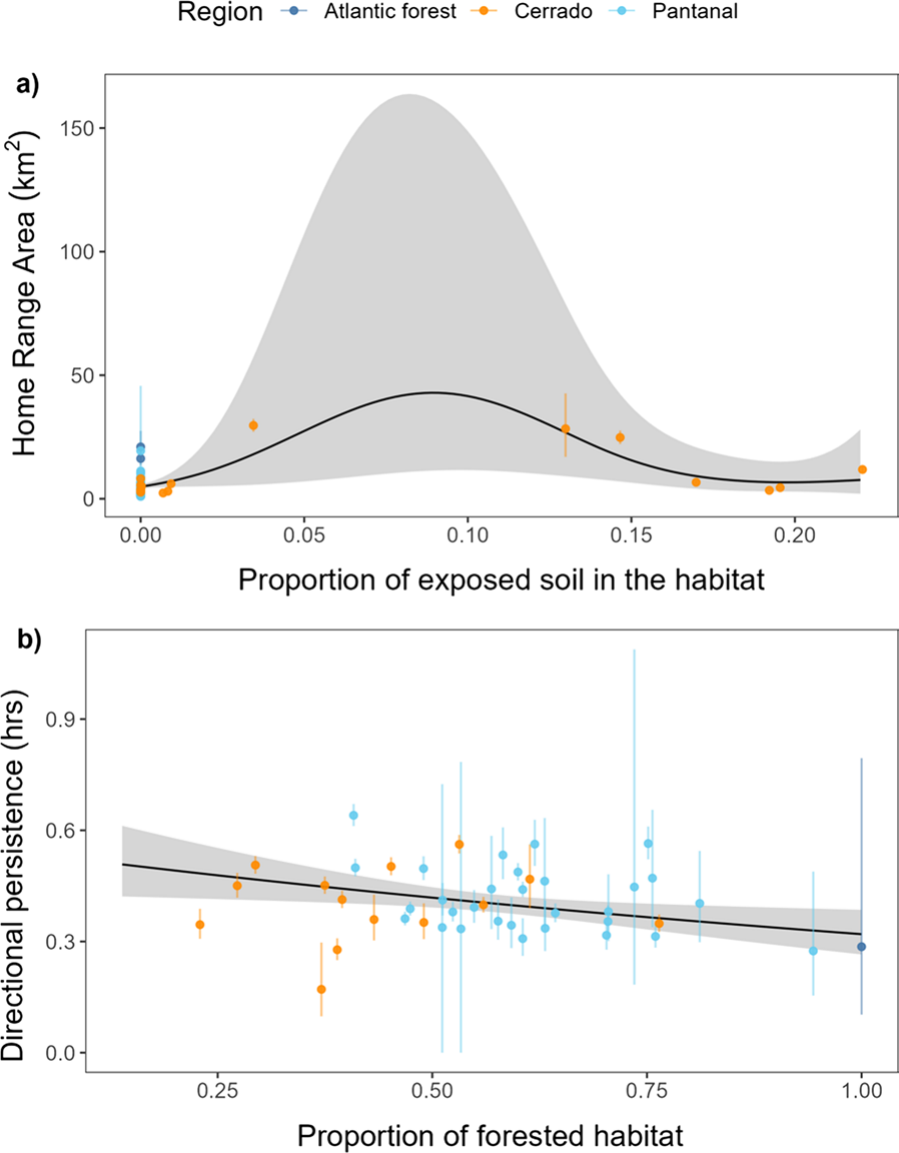
Effect of habitat types on lowland tapir space use and movement. The vertical error-bars indicate the 95% confidence intervals for the movement parameter estimates. Panel **a** depicts the estimated mean effect of exposed soil on the tapirs’ estimated home-range area. The effects of **b** forested area in a tapir’s home range on its estimated directional persistence are also shown

We also found that habitat type had little effect on HR area or average individual movement speeds. The best HR area regression model only accounted for the effect of areas of exposed soil (approximate p-value: 0.023, ${R}_{adj}^{2}$ = 0.48; Fig. 5a), while no land use types had a significant effect on an animal’s average speed. There was very little difference between the AIC of the full model (315.69, *df* = 10.18, 7 predictors and an intercept) and that of the intercept-only model (310.89, *df* = 2). However, the directional persistence term (${\tau }_{v}$) was marginally, though non-significantly lower for animals who had a higher amount of forested area (*p* = 0.093; Fig. 5b) or water (*p* = 0.025) in their home ranges. Importantly, we note here that the significant differences in directional persistence persisted even after adjusted for the increased location error in the forested areas.

**Fig. 6:**
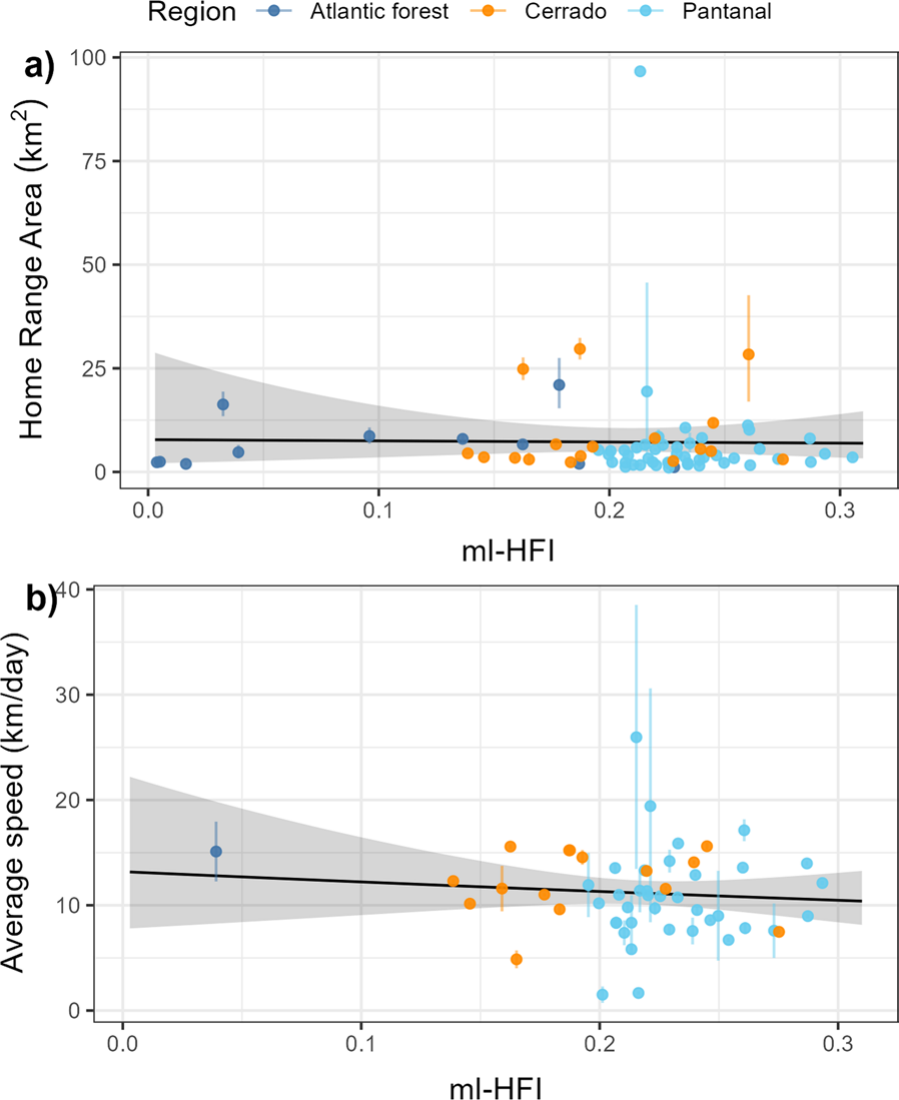
Estimated mean effect of machine-learning-based human footprint index (ml-HFI) on the tapirs’ estimated home range area and estimated average daily speed. The vertical segments indicate the 95% confidence intervals for the movement parameter estimates

HFI had no significant effect on lowland tapir home range size (*p* = 0.90; Fig. 6a), nor average daily movement speed (*p* = 0.53; Fig. 6b), nor directional persistence (*p* = 0.596, ${R}_{adj}^{2}=- 0.0184$). A tapir living in a near pristine environment (HFI = 0.004) had a home range estimate of 7.77 km^2^ (95% CI 2.12–28.6) and an average speed of 13.2 km/day (95% CI 7.8–22.1) with a directional persistence of 0.36 h (95% CI 0.16–0.78), while a tapir from the most altered habitat we monitored (HFI = 0.31) had an estimated home range area of 6.93 km^2^ (95% CI 3.36–14.30) and an average speed of 10.4 km/day (95% CI 8.3–13.2) with a directional persistence of 0.48 h (95% CI 0.34–0.68).

## Discussion

Understanding individual movement and space use requirements is a key step in conservation planning [3]. Prior to the present study, very little was known about the movement ecology of tapirs despite their vulnerable status and declining population sizes [74]. From detailed tracking of 74 tapirs collected over 22 years, we found that although individuals varied in their movement, these inter-individual differences were not explained by differences in age, sex, habitat composition, biome, nor human disturbance. Overall, human activity and land use change did not appear to significantly affect their behaviour one way or another. This contradicts patterns in large herbivores generally [16, 71], and further emphasizes the need to understand the movement ecology of target populations when designing conservation and recovery strategies.

### The ecology of lowland tapir space use

Interestingly, we found that the home range sizes and mean daily movement speeds of lowland tapirs were consistent across the three study sites. This consistency in movement was surprising as these different biomes have substantially different habitat compositions, patterns of seasonality, and productivity [47], see also Additional file 1: Appendix S1). Tapirs living in the Pantanal, for instance, occupy a near pristine ecosystem but must cope with significant seasonal flooding, whereas individuals in the Cerrado occupied an agricultural and cattle ranching mosaic with more stability across seasons. The unique requirements of these three different biomes, however, did not impact the space use and movement speed of tapirs in any statistically detectable way. Furthermore, the only pre-existing study on tapir movement found that individuals had complex home range structures, with multiple core areas of use that were established according to the distribution of patches of preferred habitat types [69]. While individuals may exhibit differential use of patchily distributed resources, we found that habitat composition had no effect on home range sizes. In addition to exhibiting little inter-individual variation in movement, variogram analysis [24] showed that tapir movement was extremely consistent over time (see also [22]. Here again, this seasonal stability in movement was interesting, especially for animals living in the Pantanal where, every year, large parts of the biome change from terrestrial into aquatic habitats and vice-versa [1]. We note though that the flooding regime in the Pantanal has been changing over the last decade and the biome is expected to become drier under the IPCC’s climate change scenarios [38].

We did find that animals with a higher proportion of forest and/or more water bodies in their home ranges had reduced directional persistence. This shows how habitat complexity can impact movement [15], with potential implications for foraging efficiency and encounter rates [5, 39, 77]. Nonetheless, these differences did not translate into patterns in tapir home range sizes and mean daily movement speeds.

### Lowland tapir movement and human disturbance

This is the first study aimed at understanding how lowland tapir space use and movement vary across differing biomes and degrees of human disturbance. Contrary to our initial expectations, and to patterns in large herbivores generally [16], human impacts on the landscape had no measurable effect on tapir movement. To put this landscape scale effect into perspective, tapirs inhabiting the Atlantic Forest, the most disturbed biome with only ca. 12–29% of habitat remaining [37, 58, 59, 66], had home ranges that were comparable in size to tapirs inhabiting the Cerrado, a biome that has lost almost 50% of its natural area (36, 45), and the Pantanal, a near pristine biome. Notably, the Lowland Tapir Conservation Action Plan published by the IUCN SSC Tapir Specialist Group (TSG) in 2007 [43], and the Lowland Tapir National Action Plan (PAN—Plano de Ação Nacional, ICMBIO—Instituto Chico Mendes de Conservação da Biodiversidade, Brazil) published in 2019 prioritize the mitigation of the impacts of small, isolated tapir populations. Population isolation thus emerges as one of the most important threats to the species’ long-term persistence. However, addressing this issue will require additional efforts as the average and maximum distances we recorded for tapir movements were substantially less than the distances between most tapir populations.

Humans are directly responsible for more than one-quarter of global terrestrial vertebrate mortality [30]. Mortality at this scale is expected to impose strong selection pressure on animal populations [53, 67]. As genotypic adaptation takes generations to occur [4], behavioral plasticity provides the most immediate response to the pressures of Human Induced Rapid Environmental Change (HIREC, [65]. The capacity for behavioural plasticity in movement and space use in response to human disturbance is especially important for long-lived, K-selected species such as tapirs [46, 60, 65] that take years to reach sexual maturity and have long inter-generational intervals [40]. Despite the key importance of behavioural adaptations in response to HIREC, tapir movement appeared to exhibit very little plasticity in response to human disturbance. The lack of any measurable response to human activity suggests that tapirs living near humans may experience increased exposure to vehicle collisions [2, 42], pesticide and environmental pollutants [20, 41, 44] and poaching [62]. Human modified habitats thus risk being ecological traps [64] for tapirs as individuals showed no detectable responses to degradations in habitat quality. Although tapir home range area and mean daily movement speed exhibited no statistically detectable response to the human footprint index, it is possible that individuals are responding to human disturbance at a finer temporal and/or spatial scale than the long-term averages that were examined here. It may also be possible that tapirs exhibit non-linear, or even binary, responses to human disturbance that were not possible to detect. Future investigation into lowland tapir behaviour in more heavily modified habitats is clearly warranted.

## Conclusions

We compared home range areas and movement behavior of lowland tapirs using telemetry data collected over 22 years across 3 biomes in southern Brazil: the Pantanal, Cerrado, and Atlantic Forest. These data represent the largest lowland tapir tracking dataset yet to be collected, with over 232,000 locations from 74 tracked individuals and fill a critical knowledge gap in lowland tapir ecology, which can contribute to long-term species management and conservation planning. Contrary to our expectations, we observed very little individual variability in lowland tapir space use and movement, and human impacts on the landscape also had no measurable effect on their movement. Lowland tapir movement behaviour thus appears to exhibit very little phenotypic plasticity. The lack of any adaptive response to anthropogenic disturbance suggests that human modified habitats risk being ecological traps for tapirs and this information should be factored into conservation actions aimed towards protecting lowland tapir populations.

## Supplementary Information

**Additional file 1:** Details on the habitat composition of the different biomes.

**Additional file 2:** Details on the R scripts used to generate the results presented in the main text.

## Data Availability

All R code and data necessary to reproduce the analyses in the main text can be found in the GitHub repository at https://github.com/StefanoMezzini/tapirs.
