# Supplementary material for: Movement ecology of vulnerable lowland tapirs between areas of varying human disturbance
Source: Mov Ecol. 2022 Mar 14;10:14. doi: 10.1186/s40462-022-00313-w (PMC8919628; doi:10.1186/s40462-022-00313-w)
Supplement: Supplementary file 1 — Additional file 1: Details on the habitat composition of the different biomes. [file 40462_2022_313_MOESM1_ESM.docx]

# Appendix S1

In this appendix we provide supporting information on the relationship between home range estimation and location estimation method (i.e., VHF vs. GPS tracking). Information on the habitat composition of the different sites is also provided.

## Home range estimates and data collection methods

The tapir location data included in this study were collected over 22 years using a mix of VHF and GPS tracking. As these two data types resulted in different sampling frequencies, it was possible for differences in autocorrelation to drive differences in the estimated home range areas (Noonan et al. 2019a). We therefore carried out a supporting analysis to ensure that there was no relationship between data collection methods and the estimated home range estimates. We found that there was no significant difference in the home range estimates between individuals who were monitored using GPS collars, VHF tracking, or a mixture of the two (GPS as the control, p-values: 0.495 for GPS and VHF, 0.739 for VHF only, see Fig. S1).


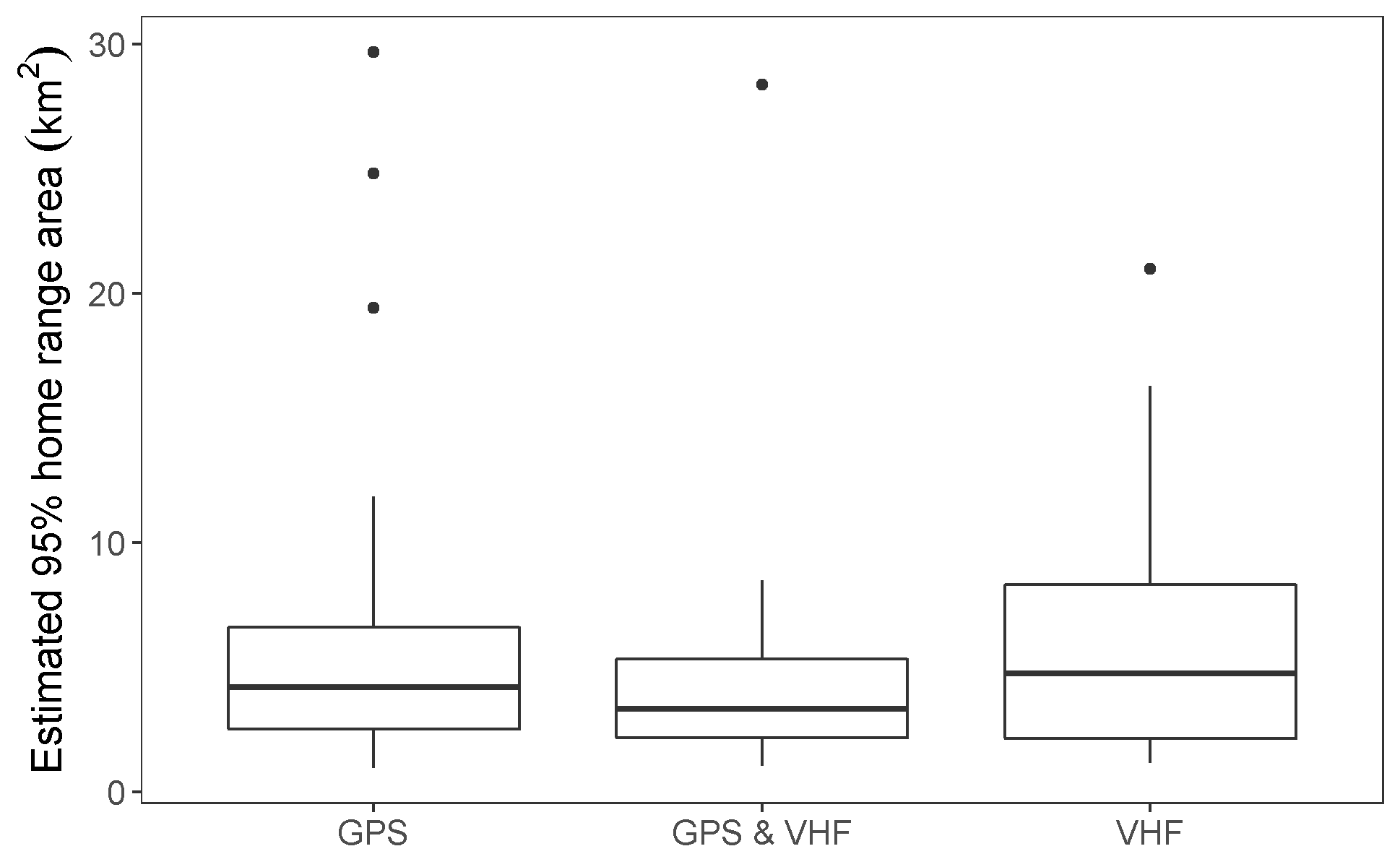


**Figure S1:** Estimated 95% home range area by tracking method. Tapirs were monitored using GPS collars, Very High Frequency (VHF) tracking, or both.

These findings suggest that any of the results presented in the main text are robust to inter-individual differences in data collection.

## Habitat composition

Here we show how the habitat composition differed between each of the three study areas. In addition, we show how the proportion of each land use type within the home range of each tapir.


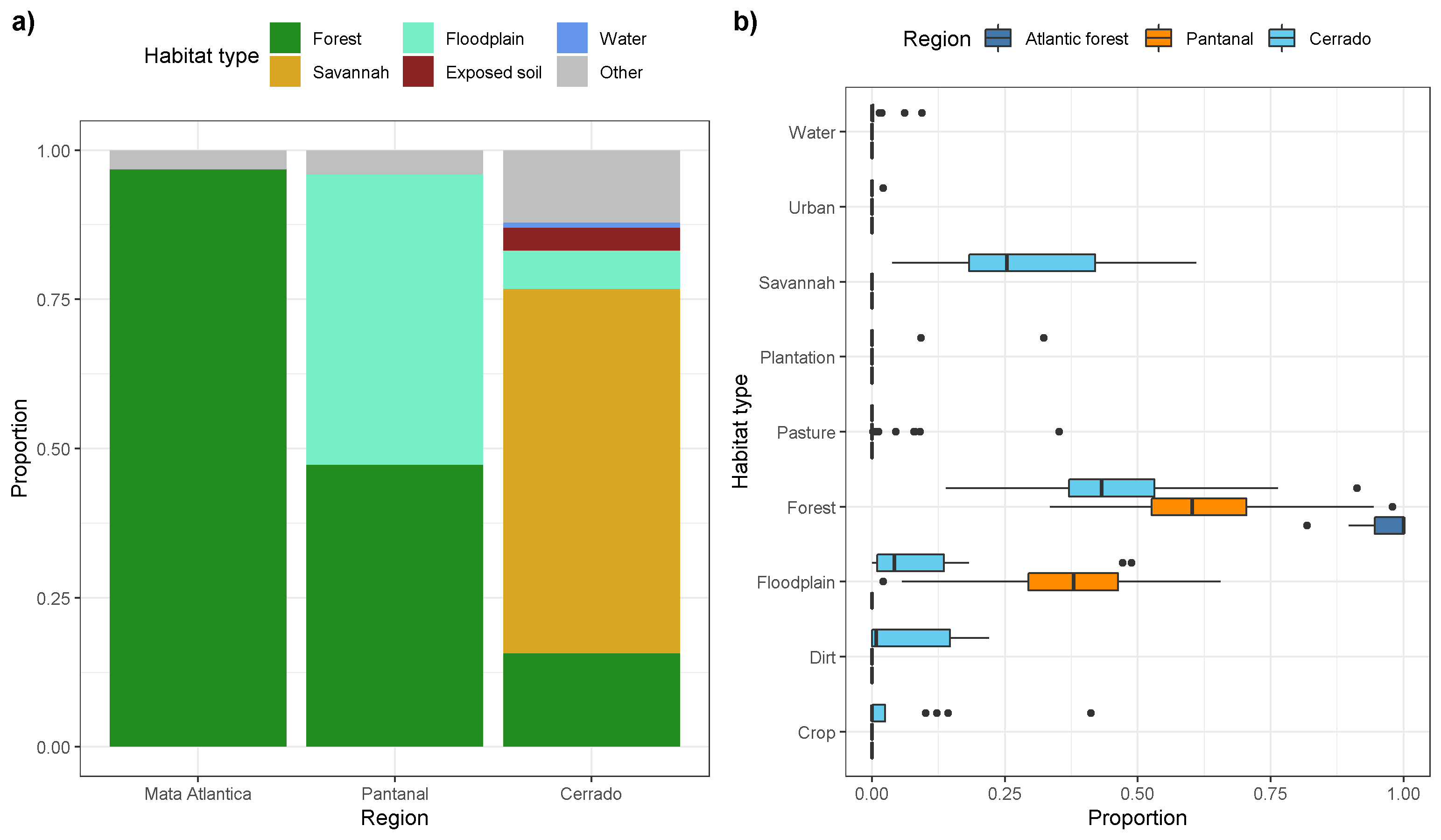


**Figure S2:** Figure depicting a) the proportion of habitat type in each of the three study areas, and b) the proportion of each land use type within the home range of each tapir.
