# Supplementary material for: Movement ecology of vulnerable lowland tapirs between areas of varying human disturbance
Source: Mov Ecol. 2022 Mar 14;10:14. doi: 10.1186/s40462-022-00313-w (PMC8919628; doi:10.1186/s40462-022-00313-w)
Supplement: Supplementary file 2 — Additional file 2: Details on the R scripts used to generate the results presented in the main text. [file 40462_2022_313_MOESM2_ESM.pdf]

Appendix - R Scripts for reproducing the results presented in the main text

EP Medici, S Mezzini, CH Fleming, JM Calabrese, MJ Noonan

2022-02-25

Contents

1 Pre-analysis setup **2**

2 Summary statistics for movement and space use parameters **3**

3 Figures **9**
3.1 ml-HFI map (figure 1) 9
3.2 AKDE 95% home range estimates (figure 2) 9
3.3 Parameter estimates using `ctmm::meta()` (figure 3) 11
3.4 Strip charts (figure 4) 15
3.5 Variation in movement across biomes and habitat composition (figure 5) 19
3.6 Variation in movement across biomes and gradients of human disturbance (figure 6) 28

4 Core home range analysis **35**
4.1 Summary statistics for movement and space use parameters 35
4.2 Figures 39

In this appendix we provide details on the analyses and R code used to generate all of the results presented in the main text. For full details on these analyses see the github repository associated with this manuscript at: https://github.com/StefanoMezzini/tapirs.

Section 1, *Pre-analysis setup*, attaches the packages needed for the analysis and imports the main dataset.

Section 2, *Summary statistics for movement and space use parameters*, shows how results were obtained for each section, following the order in which results are presented in the manuscript.

Section 3, *Figures*, shows how figures 2-6 were created (with minor changes for aesthetic purposes and ease of readability). Figure 1 is not recreated here because the final version was created in Photoshop. The code to create the figure's components can be found in the `R` script `analysis/maps.R`.

Section 4, *Core home range analysis*, repeats the sections 2 and 3 for the 50% AKDE home range estimates rather than the 95% AKDE.

1 Pre-analysis setup

```r
library('purrr')     # for functional programming
library('mgcv')      # for fitting GAMs
library('ctmm')      # for animal movement analyses
library('tidyr')     # for data wrangling
library('MuMIn')     # for model selection
library('sf')        # to work with spData maps
library('sp')        # to import spatial layers
library('raster')    # to import human footprint index raster
library('ggplot2')   # for plotting
library('ggmap')     # for maps
library('cowplot')   # for plot grids
library('gratia')    # for seq_min_max(), draw()
library('lubridate') # makes working with dates smoother
library('dplyr')     # for data wrangling
library('ncdf4')     # required for ml-HFI raster
theme_set(theme_bw())

# color palette
pal <- c('#4477AA', '#ff8c00', '#66CCEE', '#009900', '#CCBB44', '#EE6677', '#AA3377')
N <- 74 # number of tapirs

tapirs <- readRDS('../models/tapirs-final.rds') %>% # tapir data
  arrange(name) %>%
  mutate(
    # translate name
    region.lab = if_else(region.lab == 'Mata Atlantica', 'Atlantic forest', region.lab),
    # convert tau_p from seconds to days
    tau.position.est = tau.position.est / (60^2 * 24),
    tau.position.low = tau.position.low / (60^2 * 24),
    tau.position.high = tau.position.high / (60^2 * 24),
    # convert tau_v from seconds to hours
    tau.velocity.est = tau.velocity.est / (60^2),
    tau.velocity.low = tau.velocity.low / (60^2),
    tau.velocity.high = tau.velocity.high / (60^2))
```

2 Summary statistics for movement and space use parameters

```
est <- function(y, parameter = c('mean', 'lwr', 'upr')) {
  if(sum(!is.na(y)) > 2) { # if there's at least 2 observations
    # fit a Gamma GLM with only an intercept to estimate the group-level mean
    m <- gam(y ~ 1,
             family = Gamma(link = 'log'),
             method = 'REML')

    if(length(parameter) > 1) stop('Specify a single parameter.\n')

    pred <- predict(m, newdata = tibble(c = 1), se.fit = TRUE, scale = 'link') %>%
      as.data.frame() %>%
      mutate(lwr = exp(fit - 1.96 * se.fit),
             mean = exp(fit),
             upr = exp(fit + 1.96 * se.fit))
    pred[1, parameter]
  } else {NA_real_}
}

tap <-
  tapirs %>%
  bind_rows(
    bind_cols(name = c('Atlantic forest', 'Cerrado', 'Pantanal', 'Overall'),
              region.lab = c('Atlantic forest', 'Cerrado', 'Pantanal', 'Overall'),
              # 95% home range estimates
              bind_rows(
                meta(tapirs$model[tapirs$region == 'atlantica'], plot = FALSE)[1, ],
                meta(tapirs$model[tapirs$region == 'cerrado'], plot = FALSE)[1, ],
                meta(tapirs$model[tapirs$region == 'pantanal'], plot = FALSE)[1, ],
                meta(tapirs$model, plot = FALSE)[1, ]) %>%
                rename(area.low = low, area.est = est, area.high = high),
              # regional estimates
              group_by(tapirs, region) %>%
                summarize(tau.position.e = est(tau.position.est, 'mean'),
                          tau.position.low = est(tau.position.est, 'lwr'),
                          tau.position.high = est(tau.position.est, 'upr'),
                          tau.velocity.e = est(tau.velocity.est, 'mean'),
                          tau.velocity.low = est(tau.velocity.est, 'lwr'),
                          tau.velocity.high = est(tau.velocity.est, 'upr'),
                          speed.e = est(speed.est, 'mean'),
                          speed.low = est(speed.est, 'lwr'),
                          speed.high = est(speed.est, 'upr')) %>%
                rename(tau.position.est = tau.position.e,
                       tau.velocity.est = tau.velocity.e,
                       speed.est = speed.e) %>%
                bind_rows(
                  tapirs %>%
                    summarize(tau.position.est = est(tapirs$tau.position.est, 'mean'),
                              tau.position.low = est(tapirs$tau.position.est, 'lwr'),
                              tau.position.high = est(tapirs$tau.position.est, 'upr'),
                              tau.velocity.est = est(tapirs$tau.velocity.est, 'mean'),
                              tau.velocity.low = est(tapirs$tau.velocity.est, 'lwr'),
```

```
                              tau.velocity.high = est(tapirs$tau.velocity.est, 'upr'),
                              speed.est = est(tapirs$speed.est, 'mean'),
                              speed.low = est(tapirs$speed.est, 'lwr'),
                              speed.high = est(tapirs$speed.est, 'upr'))))) %>%
  mutate(name = factor(name, levels = c(unique(tapirs$name), 'Atlantic forest',
                                         'Cerrado', 'Pantanal', 'Overall')),
         region.lab = factor(region.lab,
                             levels = c('Atlantic forest', 'Cerrado', 'Pantanal',
                                        'Overall')),
         average = if_else(name %in% c('Atlantic forest','Cerrado','Pantanal','Overall'),
                           'Group mean', 'Individual') %>%
           factor(levels = c('Individual', 'Group mean'))) %>%
  select(region.lab, name, area.low:area.high, tau.position.est:tau.position.low,
         speed.est:tau.velocity.low, average)
```

```
# est = estimated mean; low = lower 95% CI limit, high = upper 95% CI limit
tap %>%
  filter(average == 'Group mean') %>% # only group means
  select(-c(average, region.lab)) %>%
  relocate(name) %>% # move to be first column
  mutate(across(where(is.double), \(x) round(x, 2))) %>% # round to 2 decimals
  t() %>% # transpose to keep within page limits
  knitr::kable(caption = "Habitat-specific and overall means and 95% confidence intervals for the tapir
```

Table 1: Habitat-specific and overall means and 95% confidence intervals for the tapirs' movement parameter estimates.

name	Atlantic forest	Cerrado	Pantanal	Overall
area.low	4.38	7.65	4.70	6.53
area.est	10.29	13.38	5.94	8.31
area.high	20.74	21.80	7.40	10.42
tau.position.est	0.54	0.66	0.80	0.72
tau.position.high	1.54	1.08	1.76	1.25
tau.position.low	0.19	0.40	0.36	0.42
speed.est	NA	12.14	10.67	11.17
speed.high	NA	13.86	12.21	12.35
speed.low	NA	10.64	9.33	10.11
tau.velocity.est	NA	0.40	0.46	0.44
tau.velocity.high	NA	0.46	0.57	0.51
tau.velocity.low	NA	0.35	0.37	0.38

The overall mean 95% home range estimate was 8.31 km^2 with CI (6.53, 10.42) km^2 (see Table 1 above).

```
range(tapirs$area.est) %>% round(digits = 1)
```

```
## [1]  1.0 29.7
```

The range in home range area estimates was 1.0 - 29.7 km^2.

```
round(range(tapirs$tau.position.est, na.rm = TRUE), 2)
```

```
## [1]  0.05 12.76
```

The range in range crossing time was 0.05-12.76 days.

The overall average directional persistence was 0.44 hours with a 95% CI of (0.38, 0.51) hours (see Table 1 above).

```
round(range(tapirs$tau.velocity.est, na.rm = TRUE), 2)
```

```
## [1] 0.17 1.88
```

The range in mean directional persistence timescale was 0.17-1.88 hours.

The overall average speed was 11.2 km/day with a 95% CI of (10.1, 12.3) km/day (see Table 1 above).

```
range(tapirs$speed.est, na.rm = TRUE) %>% round(2)
```

```
## [1]  1.51 25.96
```

The range in average daily speed was 1.51 - 25.96 km/day.

When we first submitted the manuscript using `ctmm` version 0.6.1, `ctmm::meta()` could only produce estimates for home ranges. Thus, we used Gamma GLMs fit via `mgcv::gam()` to estimate differences in mean daily movement speed between animals of different ages and sexes:

```
# Differences in movement speed between animals of different ages and sexes
m.speed.sex <- gam(speed.est ~ sex,
                   family = Gamma('log'),
                   data = tapirs,
                   method = 'REML')
m.speed.age <- gam(speed.est ~ adult,
                   family = Gamma('log'),
                   data = tapirs,
                   method = 'REML')
# model summaries
summary(m.speed.sex)
```

```
##
## Family: Gamma
## Link function: log
##
## Formula:
## speed.est ~ sex
##
## Parametric coefficients:
##             Estimate Std. Error t value Pr(>|t|)
## (Intercept)  2.35276    0.06872  34.235   <2e-16 ***
## sexMALE      0.12420    0.10006   1.241     0.22
## ---
## Signif. codes:  0 '***' 0.001 '**' 0.01 '*' 0.05 '.' 0.1 ' ' 1
##
##
## R-sq.(adj) =  0.00946   Deviance explained = 2.16%
## -REML = 156.04  Scale est. = 0.13224   n = 53
```

```
summary(m.speed.age)
```

```
##
## Family: Gamma
## Link function: log
##
## Formula:
## speed.est ~ adult
```

```
##
## Parametric coefficients:
##             Estimate Std. Error t value Pr(>|t|)
## (Intercept)   2.2532     0.0928  24.281   <2e-16 ***
## adultYes      0.2168     0.1096   1.978   0.0533 .
## ---
## Signif. codes:  0 '***' 0.001 '**' 0.01 '*' 0.05 '.' 0.1 ' ' 1
##
##
## R-sq.(adj) =  0.0454   Deviance explained = 5.18%
## -REML = 155.11  Scale est. = 0.12917   n = 53
```

```
# predictions
tibble(Sex = c('FEMALE', 'MALE'),
       bind_cols(predict(m.speed.sex, tibble(sex = Sex), se.fit = TRUE))) %>%
  mutate(est = round(exp(fit), 2),
         lwr = round(exp(fit - 1.96 * se.fit), 2),
         upr = round(exp(fit + 1.96 * se.fit), 2))
```

```
## # A tibble: 2 x 6
##   Sex      fit se.fit   est   lwr   upr
##   <chr>  <dbl>  <dbl> <dbl> <dbl> <dbl>
## 1 FEMALE  2.35 0.0687  10.5  9.19  12.0
## 2 MALE    2.48 0.0727  11.9 10.3   13.7
```

```
tibble(Adult = c('Yes', 'No'),
       bind_cols(predict(m.speed.age, tibble(adult = Adult), se.fit = TRUE))) %>%
  mutate(est = round(exp(fit), 2),
         lwr = round(exp(fit - 1.96 * se.fit), 2),
         upr = round(exp(fit + 1.96 * se.fit), 2))
```

```
## # A tibble: 2 x 6
##   Adult   fit se.fit   est   lwr   upr
##   <chr> <dbl>  <dbl> <dbl> <dbl> <dbl>
## 1 Yes    2.47 0.0583 11.8  10.6   13.2
## 2 No     2.25 0.0928  9.52  7.94  11.4
```

There was no evidence that daily speed differed between sexes (females: 10.5 km/day, 95% CI: 9.19 - 12.0; males: 11.9 km/day; 95% CI: 10.3 - 13.7, $p = 0.22$, 4a), nor between age groups (adults: 11.8 km/day, 95% CI: 10.6 - 13.2; sub-adults: 9.52 km/day, 95% CI: 7.94 - 11.4; $p = 0.053$, Fig. 4b).

```
# by sex
meta(list(female = filter(tapirs, sex == 'FEMALE')$akde,
          male = filter(tapirs, sex == 'MALE')$akde),
     plot = FALSE, verbose = TRUE) # verbose output with CIs
```

```
## * Sub-population female

##                           <U+0394>AICc
## inverse-Gaussian        0.000
## Dirac-d              7367.412

## * Sub-population male

##                           <U+0394>AICc
## inverse-Gaussian        0.000
## Dirac-d              7240.514

## * Joint population
```

```
##                           <U+0394>AICc
## inverse-Gaussian           0.00
## Dirac-d                14778.71
```

```
## * Joint population versus sub-populations (best models)
```

```
##                           <U+0394>AICc
## Joint population        0.000000
## Sub-population          4.035557
```

```
## $female
##                     low      est     high
## mean (km²)   4.5262477 6.113163 8.068988
## CoV² (RVAR) 0.4615142 0.861599 1.384494
## CoV  (RSTD) 0.6857439 0.936962 1.187722
##
## $male
##                     low       est     high
## mean (km²)   4.0316207 5.4605379 7.232389
## CoV² (RVAR) 0.3590259 0.7091032 1.176317
## CoV  (RSTD) 0.6057262 0.8512718 1.096417
##
## $`mean ratio`
## , , low
##
##             /female     /male
## female/ 1.0000000 0.7026962
## male/   0.5608837 1.0000000
##
## , , est
##
##             /female    /male
## female/ 1.0000000 1.094326
## male/   0.8736009 1.000000
##
## , , high
##
##            /female    /male
## female/ 1.000000 1.629906
## male/   1.300086 1.000000
```

```
# by age group
meta(list(subadult = filter(tapirs, adult == 'No')$akde,
          adult = filter(tapirs, adult == 'Yes')$akde),
     plot = FALSE, verbose = TRUE)
```

```
## * Sub-population subadult
```

```
##                           <U+0394>AICc
## inverse-Gaussian          0.000
## Dirac-d                4785.763
```

```
## * Sub-population adult
```

```
##                           <U+0394>AICc
## inverse-Gaussian          0.000
## Dirac-d                9841.229
```

```
## * Joint population
```

```
##                        <U+0394>AICc
## inverse-Gaussian         0.00
## Dirac-d              14778.71
```

```
## * Joint population versus sub-populations (best models)
```

```
##                        <U+0394>AICc
## Sub-population        0.00000
## Joint population      3.08865
```

```
## $subadult
##                   low      est      high
## mean (km²)  3.8693253 6.985177 11.648649
## CoV² (RVAR) 0.5763867 1.674818  3.348693
## CoV  (RSTD) 0.7764644 1.323574  1.871554
##
## $adult
##                   low       est      high
## mean (km²)  4.3936522 5.3698397 6.4954770
## CoV² (RVAR) 0.3073040 0.5063390 0.7542649
## CoV  (RSTD) 0.5578842 0.7161115 0.8740210
##
## $`mean ratio`
## , , low
##
##           /subadult    /adult
## subadult/ 1.0000000 0.6457532
## adult/    0.3654868 1.0000000
##
## , , est
##
##           /subadult   /adult
## subadult/ 1.0000000 1.287811
## adult/    0.7053565 1.000000
##
## , , high
##
##           /subadult  /adult
## subadult/  1.000000 2.17904
## adult/     1.321562 1.00000
```

Using `ctmm::meta()`, we found that home range area was not significantly different ($\alpha = 0.05$) between sexes (females: mean = 6.11, 95% CI = (4.53, 8.07); males: mean = 5.46, 95% CI = (4.03, 7.23); LRT for female $>$ male = 1.09, 95% CI = (0.70, 1.63)) nor between age groups (sub-adults: mean = 6.99, 95% CI = (3.87, 11.65); adults: mean = 5.37, 95% CI = (4.39, 6.50); LRT for sub-adult $>$ adult = 1.29, 95% CI = (0.65, 2.18)).

3 Figures

3.1 ml-HFI map (figure 1)

Figure 1 is not recreated here because the final version was created in Photoshop. The code to create the figure's components can be found in the R script `analysis/maps.R`.

3.2 AKDE 95% home range estimates (figure 2)

```r
tapirs <- tapirs %>%
  mutate(proj = map(data, function(d) CRS(d@info$projection)),
         akde.df =
           map(1:N,
               function(i)
                 SpatialPolygonsDataFrame.UD(akde[[i]],
                                             proj4string = proj[[i]]) %>%
                 spTransform(CRS("+proj=longlat")) %>%
                 fortify()))

atl.akdes <- bind_rows(tapirs$akde.df) %>% filter(grepl('AF_', group))
cer.akdes <- bind_rows(tapirs$akde.df) %>% filter(grepl('CE_', group))
pan.akdes <- bind_rows(tapirs$akde.df) %>% filter(grepl('PA_', group))

plot.akde <- function(reg) {
  if(reg == 'Atlantic Forest') {
    r <- 'AF_'
    ZOOM <- 11
  } else if(reg == 'Cerrado'){
    r <- 'CE_'
    ZOOM <- 10
  } else if(reg == 'Pantanal') {
    r <- 'PA_'
    ZOOM <- 12
  } else {
    stop('invalid region name')
  }
  akdes <- bind_rows(tapirs$akde.df) %>%
    filter(grepl(r, group), grepl('est', group))

  # box expansion factor
  k <- case_when(reg == 'Pantanal' ~ 0.6,
                 reg == 'Cerrado' ~ 0.1,
                 reg == 'Atlantic Forest' ~ 0.5)

  BOX <- akdes %>%
    summarize(left = min(long),
              bottom = min(lat),
              right = max(long),
              top = max(lat)) %>%

    # expand boxes slightly
    mutate(left = left - k * (right - left),
           right = right + k * (right - left),
           bottom = bottom - 0.1 * (top - bottom),
```

```
          top = top + 0.1 * (top - bottom)) %>%
    unlist()

  MAP <- get_map(BOX, zoom = ZOOM,
                 maptype = "satellite",
                 style="element:labels|visibility:off|
                    style=feature:administrative.land_parcel|
                    visibility:off|style=feature:administrative.neighborhood
                    |visibility:off")

  TITLE <- case_when(reg == 'Atlantic Forest'~ 'a)',
                     reg == 'Cerrado' ~ 'b)',
                     reg == 'Pantanal' ~ 'c)')

  ggmap(MAP, darken = c(0.3, "white")) +
    geom_polygon(aes(x = long, y = lat, fill = group), color = 'black',
                 alpha = 0.5, akdes, lwd = 0.15) +
    scale_fill_viridis_d('Human Footprint Index', option = 'B') +
    labs(x = 'Longitude', y = 'Latitude', title = TITLE) +
    theme(panel.border = element_rect(colour = 'black', fill = 'transparent'),
          legend.position = 'none')
}

plot_grid(plot.akde('Atlantic Forest'), plot.akde('Cerrado'), plot.akde('Pantanal'),
          ncol = 2) # used ncol = 1 for figure in the main text
```

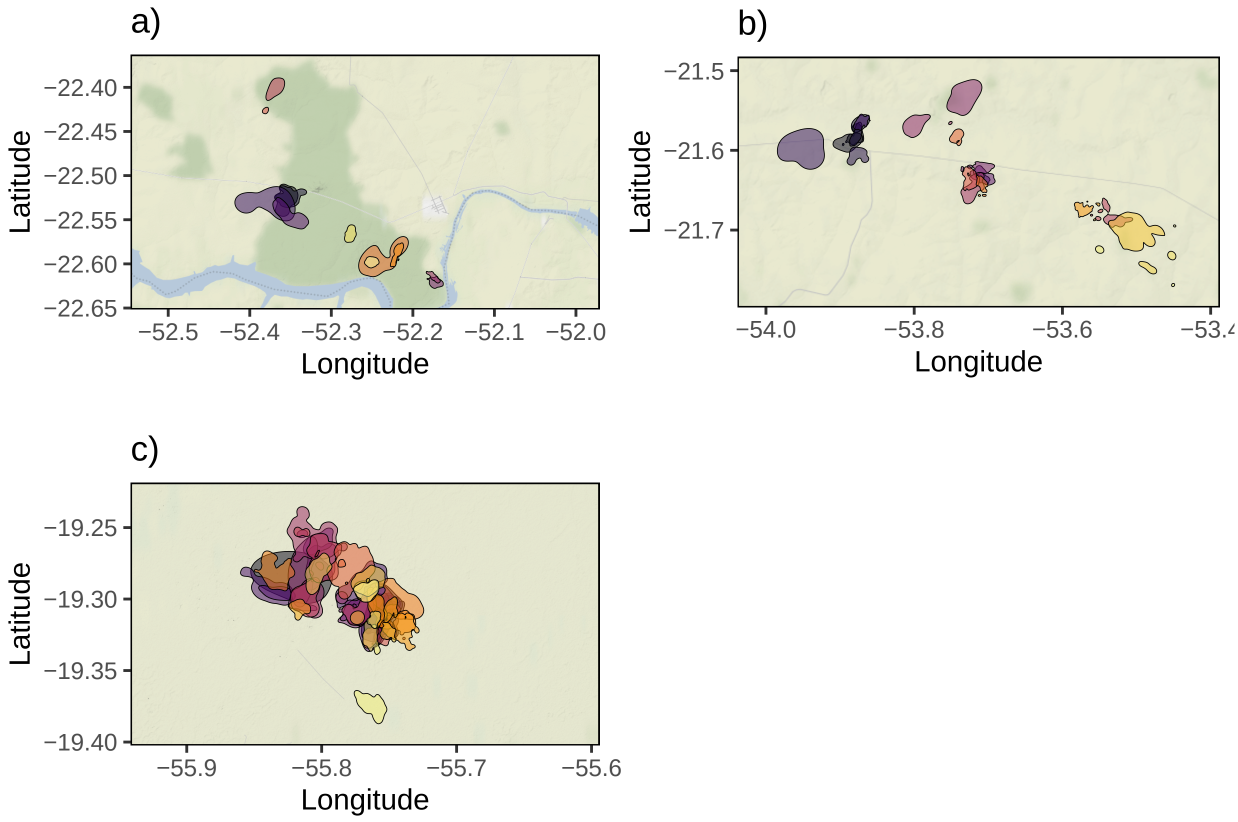

3.3 Parameter estimates using `ctmm::meta()` (figure 3)

```
# 3a) meta() of areas
ggplot(tap) +
  geom_segment(aes(x = area.low, xend = area.high, y = name, yend = name,
                   color = region.lab), lwd = 2) +
  geom_point(aes(x = area.est, y = name, shape = average), col = 'black', size = 1.2) +
  geom_point(aes(x = area.est, y = name, shape = average), col = 'white', size = 0.7) +
  scale_shape_manual(element_blank(), values = c(19, 17)) +
  scale_color_manual('Region', values = c(pal[1:3], 'black')) +
  scale_y_discrete(limits = rev,
                   labels = c('Means', 'Pantanal', 'Cerrado', 'Atlantic forest'),
                   breaks = c('Atlantic forest', 'PA_33_GABRIELA', 'CE_15_KURUKA',
                              'AF_14_JAMESBOND')) +
  labs(x = bquote('Estimated 95% home range area'~(km^2)),
       y = NULL)
```

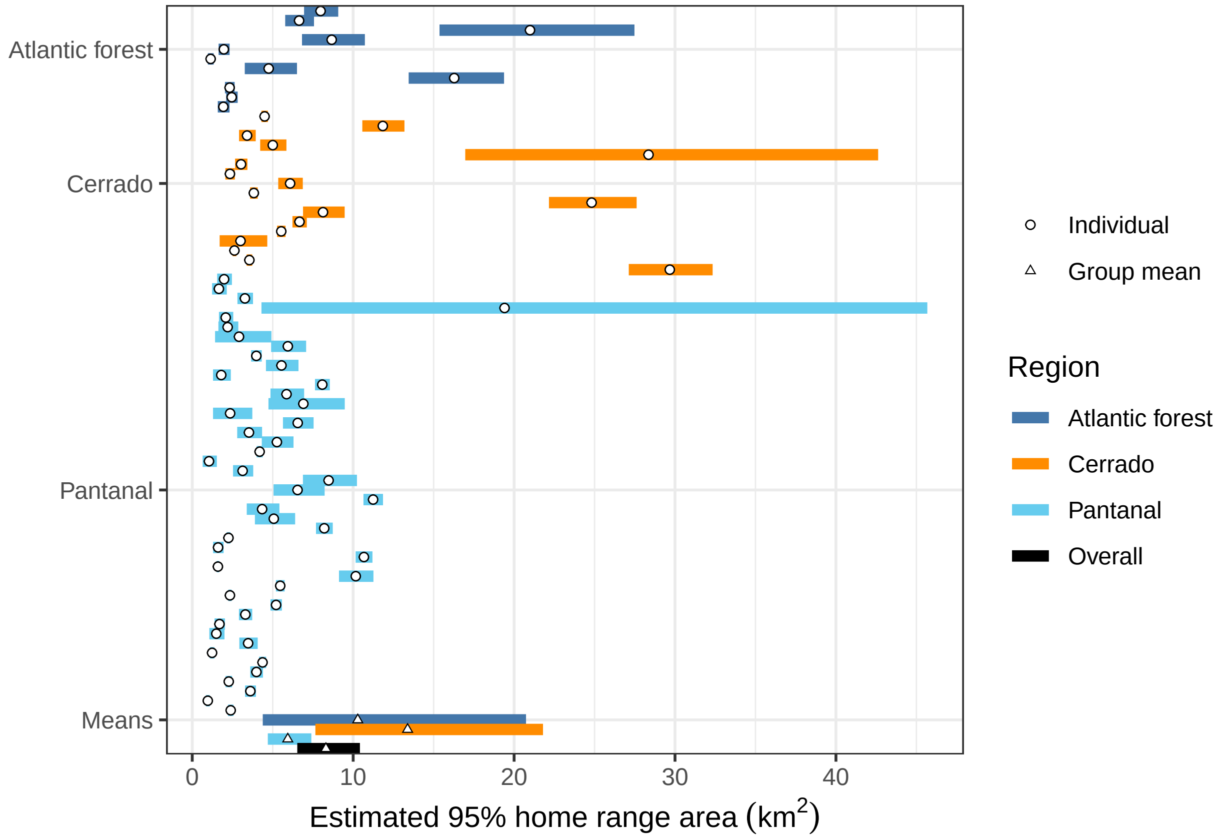

```r
# 3b) home range crossing times
ggplot(tap) +
  geom_segment(aes(x = tau.position.low,
                   xend = tau.position.high, y = name, yend = name,
                   color = region.lab), lwd = 2) +
  geom_point(aes(x = tau.position.est, y = name, shape = average),
             col = 'black', size = 1.2) +
  geom_point(aes(x = tau.position.est, y = name, shape = average),
             col = 'white', size = 0.7) +
  scale_shape_manual(element_blank(), values = c(19, 17)) +
  scale_color_manual('Region', values = c(pal[1:3], 'black')) +
  scale_y_discrete(limits = rev,
                   labels = c('Means', 'Pantanal', 'Cerrado', 'Atlantic forest'),
                   breaks = c('Atlantic forest', 'PA_33_GABRIELA', 'CE_15_KURUKA',
                              'AF_14_JAMESBOND')) +
  labs(x = 'Range crossing time (days)', y = NULL)
```

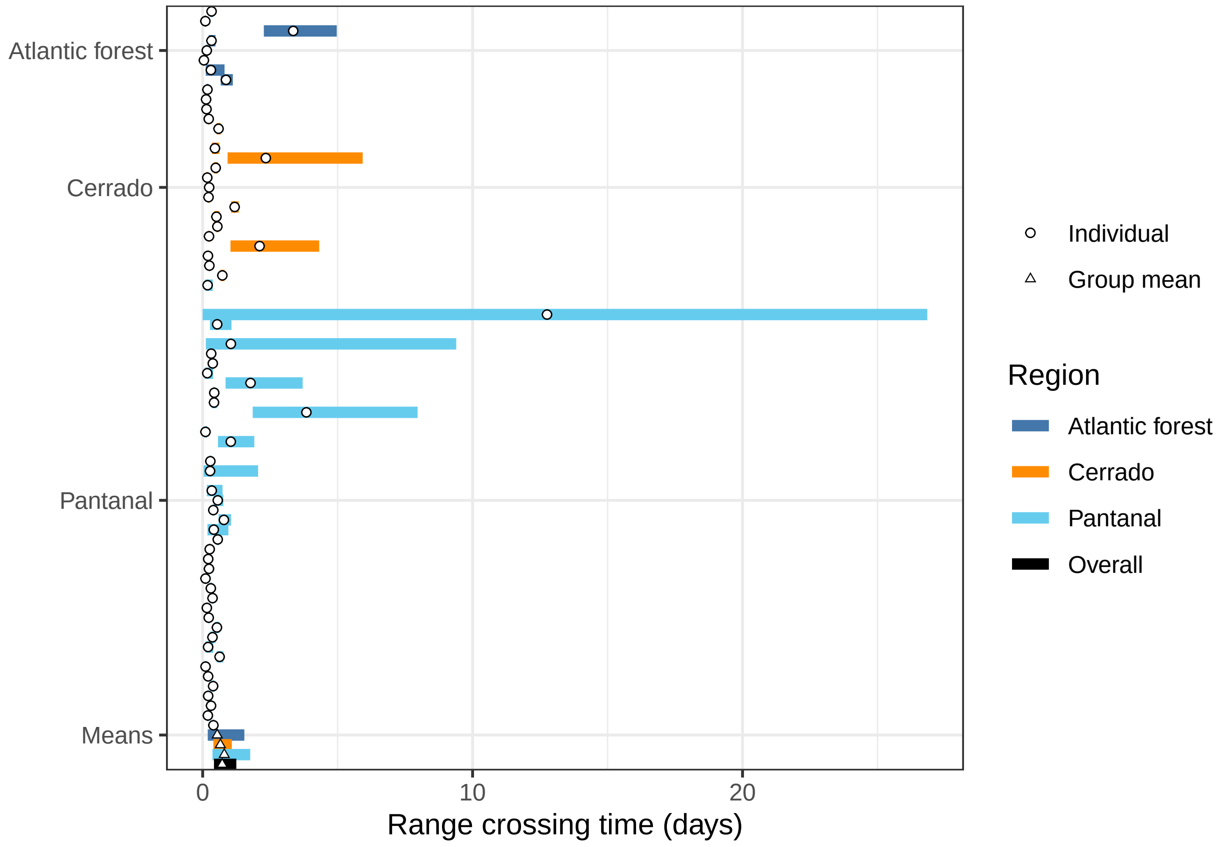

```r
# 3c) velocity autocorrelation timescale
ggplot(tap) +
  geom_segment(aes(x = tau.velocity.low, xend = tau.velocity.high, y = name, yend = name,
                   color = region.lab), lwd = 2) +
  geom_point(aes(x = tau.velocity.est, y = name, shape = average),
             col = 'black', size = 1.2) +
  geom_point(aes(x = tau.velocity.est, y = name, shape = average),
             col = 'white', size = 0.7) +
  scale_shape_manual(element_blank(), values = c(19, 17)) +
  scale_color_manual('Region', values = c(pal[1:3], 'black')) +
  scale_y_discrete(limits = rev,
                   labels = c('Means', 'Cerrado', 'Pantanal', 'Atlantic forest'),
                   breaks = c('Atlantic forest', 'CE_15_KURUKA',
                              'PA_33_GABRIELA', 'AF_14_JAMESBOND')) +
  labs(x = 'Directional persistence timescale (hours)', y = NULL)
```

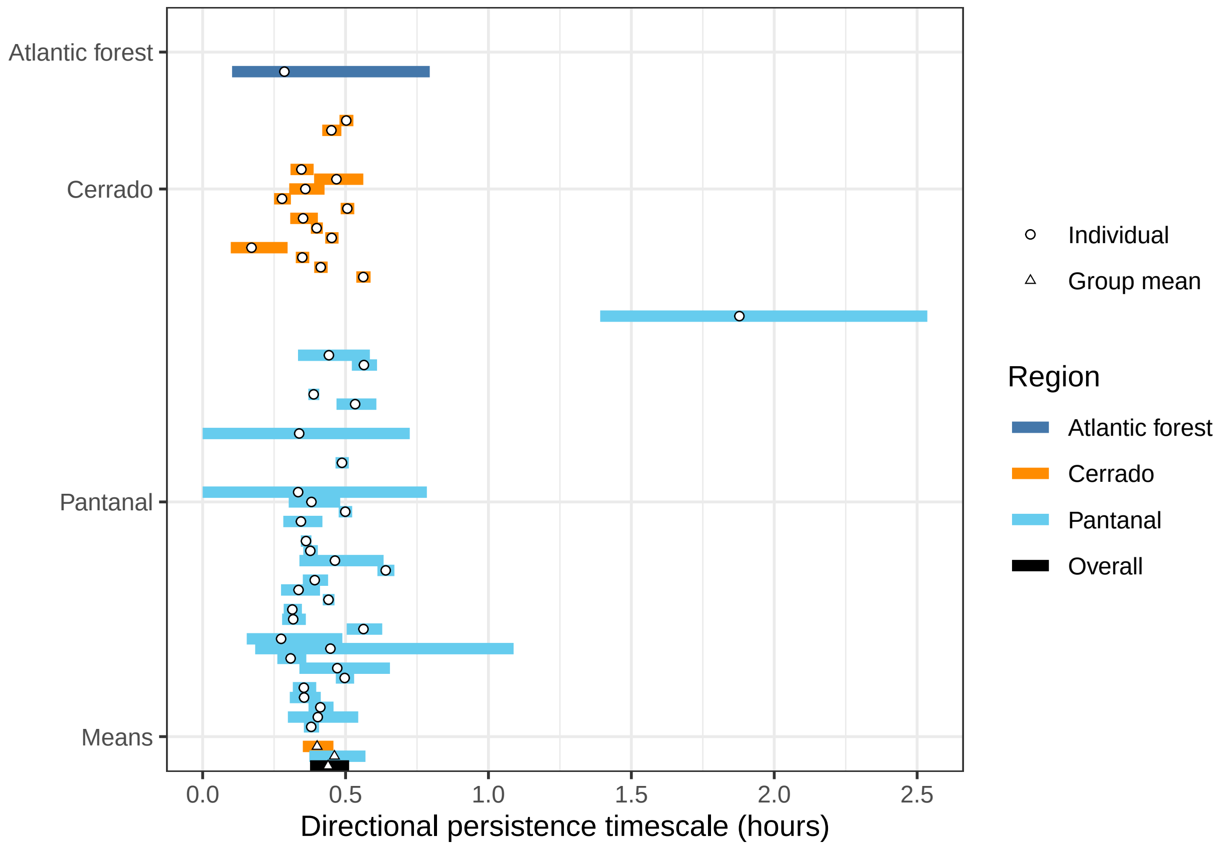

```r
# 3d) mean movement speeds
ggplot(tap) +
  geom_segment(aes(x = speed.low, xend = speed.high,
                   y = name, yend = name, color = region.lab), lwd = 2) +
  geom_point(aes(x = speed.est, y = name, shape = average), col = 'black', size = 1.2) +
  geom_point(aes(x = speed.est, y = name, shape = average), col = 'white', size = 0.7) +
  scale_shape_manual(element_blank(), values = c(19, 17)) +
  scale_color_manual('Region', values = c(pal[1:3], 'black')) +
  scale_y_discrete(limits = rev,
                   labels = c('Atlantic forest', 'Pantanal', 'Cerrado', 'Means'),
                   breaks = c('AF_17_ESPERTA', 'PA_33_GABRIELA', 'CE_15_KURUKA',
                              'Atlantic forest')) +
  labs(x = 'Estimated average speed (km/day)', y = NULL)
```

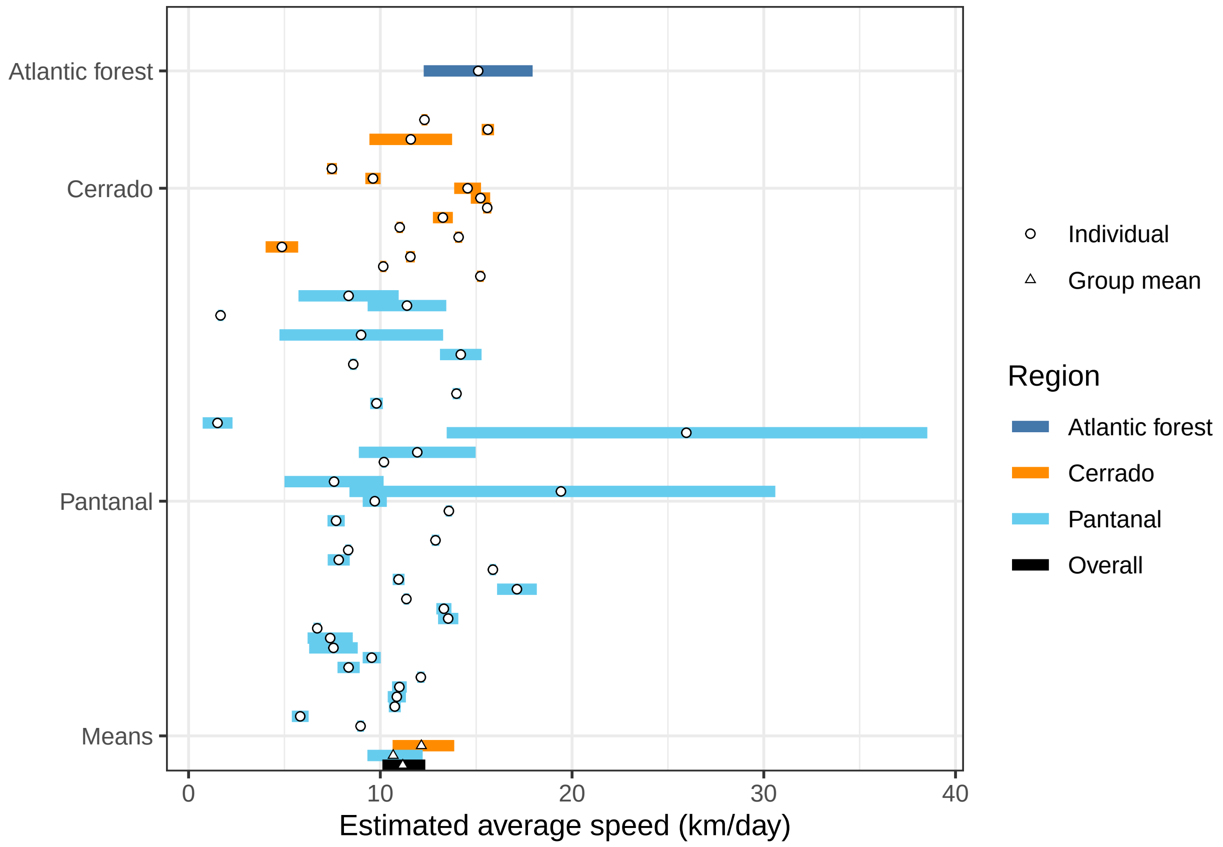

3.4 Strip charts (figure 4)

```r
# import data
tapirs <- tapirs %>%
  mutate(sex = if_else(sex == 'FEMALE', 'Female', 'Male'),
         adult = if_else(adult == 'Yes', 'Adult', 'Young'),
         name = factor(name,
                       levels = c(unique(name), 'Atlantic forest', 'Pantanal', 'Cerrado',
                                  'Overall')),
         sex_r = paste(sex, region.lab, sep = '_') %>%
           factor(levels = c('Female_Atlantic forest', 'Female_Cerrado','Female_Pantanal',
                             'Male_Atlantic forest', 'Male_Cerrado', 'Male_Pantanal')),
         adult_r = paste(adult, region.lab, sep = '_') %>%
           factor(levels = c('Adult_Atlantic forest', 'Adult_Cerrado', 'Adult_Pantanal',
                             'Young_Atlantic forest', 'Young_Cerrado', 'Young_Pantanal')))

# means and CIs
m_speed_sex <- gam(speed.est ~ sex + region.lab,
                   family = Gamma('log'),
                   data = tapirs,
                   method = 'REML')
m_speed_age <- gam(speed.est ~ adult + region.lab,
                   family = Gamma('log'),
                   data = tapirs,
                   method = 'REML')

# predictions ---
## speed, sex
summ_ss <-
  expand_grid(sex = unique(tapirs$sex),
              region.lab = unique(tapirs$region.lab)) %>%
  tibble(bind_cols(predict(m_speed_sex, se.fit = TRUE,
                           tibble(sex = sex, region.lab = region.lab)))) %>%
  mutate(sex_r = paste(sex, region.lab, sep = '_') %>%
           factor(levels = levels(tapirs$sex_r)),
         est = round(exp(fit), 2),
         lwr = round(exp(fit - 1.96 * se.fit), 2),
         upr = round(exp(fit + 1.96 * se.fit), 2))
## speed, adult
summ_sa <-
  expand_grid(adult = unique(tapirs$adult),
              region.lab = unique(tapirs$region.lab)) %>%
  tibble(bind_cols(predict(m_speed_age, se.fit = TRUE,
                           tibble(adult = adult, region.lab = region.lab)))) %>%
  mutate(adult_r = paste(adult, region.lab, sep = '_') %>%
           factor(levels = levels(tapirs$adult_r)),
         est = round(exp(fit), 2),
         lwr = round(exp(fit - 1.96 * se.fit), 2),
         upr = round(exp(fit + 1.96 * se.fit), 2))

## function to extract home ranges
hr_estimates <- function(REGION, SEX = NA, ADULT = NA, level.ud) {
  if(! is.na(SEX)) {
    d <- tapirs %>% filter(sex == SEX) # filter to a single sex
```

```r
  } else {
    d <- tapirs %>% filter(adult == ADULT) # filter to a single sex
  }

  d <-
    d %>%
    filter(region.lab == REGION) %>% # filter to a single region
    pull(akde) %>%
    meta(plot = FALSE, level.UD = level.ud) %>%
    as_tibble() %>%
    slice(1) %>%
    rename(lwr = low, upr = high) %>%
    mutate(region.lab = REGION, sex = SEX, adult = ADULT)

  if(! is.na(SEX)) {
    d <- d %>% mutate(sex_r = paste(sex, region.lab, sep = '_') %>%
                        factor(levels = levels(tapirs$sex_r)))
  } else {
    d <- d %>% mutate(adult_r = paste(adult, region.lab, sep = '_') %>%
                        factor(levels = levels(tapirs$adult_r)))
  }
}

## home range, sex
summ_as <-
  expand_grid(sex = unique(tapirs$sex),
              region.lab = unique(tapirs$region.lab)) %>%
  mutate(bind_cols(map2_dfr(region.lab, sex,
                            \(x, y) hr_estimates(REGION = x, SEX = y, level.ud = 0.95))))

## home range, adult
summ_aa <-
  expand_grid(adult = unique(tapirs$adult),
              region.lab = unique(tapirs$region.lab)) %>%
  mutate(bind_cols(map2_dfr(region.lab, adult,
                            \(x, y) hr_estimates(REGION = x, ADULT = y, level.ud=0.95))))

# function for summary plots ----
summary_plot <- function(Y, group = c('sex', 'adult')) {

  x <- paste0(group, '_r')
  summarized <- get(paste0('summ_', substr(Y, 1, 1), substr(x, 1, 1)))

  if(length(group) > 1) stop('Select only one group.')

  colnames(tapirs)[grepl(x, colnames(tapirs))] <- 'x'
  colnames(tapirs)[grepl(Y, colnames(tapirs))] <- 'y'
  colnames(summarized)[grepl(x, colnames(summarized))] <- 'x'

  p <-
    ggplot(tapirs) +
    # CIs
    geom_errorbar(aes(x, ymin = lwr, ymax = upr, color = region.lab),
```

```r
                  summarized, lwd = 3, width = 0, alpha = 0.5) +
    # data
    geom_jitter(aes(x, y, color = region.lab), shape = 4, width = 0.2, size = 2,
                na.rm = TRUE) +
    # means
    geom_point(aes(x, est), summarized, size = 2) +
    geom_point(aes(x, est), summarized, color = 'white') +

    # theme
    scale_fill_manual('Region', values = pal, aesthetics = c('fill', 'color')) +
    theme(legend.position = 'none')

  # add appropriate labels depending on grouping
  if(group == 'sex') {
    p <- p +
      scale_x_discrete(NULL, breaks = c('Female_Cerrado', 'Male_Cerrado'),
                       labels = c('Female', 'Male'))
  } else {
    p <- p +
      scale_x_discrete(NULL, breaks = c('Adult_Cerrado', 'Young_Cerrado'),
                       labels = c('Adult', 'Young'))
  }

  # add appropriate y label
  if(Y == 'speed.est') {
    p + scale_y_continuous('Speed (km/day)')
  } else {
    p + scale_y_continuous(expression(Home~range~(km^2)))
  }
}

# summary plots ----
spe_s <- summary_plot(group = 'sex', Y = 'speed.est')
spe_a <- summary_plot(group = 'adult', Y = 'speed.est')
hr_s <- summary_plot(group = 'sex', Y = 'area.est')
hr_a <- summary_plot(group = 'adult', Y = 'area.est')
```

```r
plot_grid(get_legend(spe_s +
                       theme(legend.position = 'top')),
          plot_grid(spe_s, spe_a, hr_s, hr_a, ncol = 2, label_y = 1.08,
                    labels = c('a)', 'b)', 'c)', 'd)')),
          ncol = 1, rel_heights = c(0.1, 1))
```

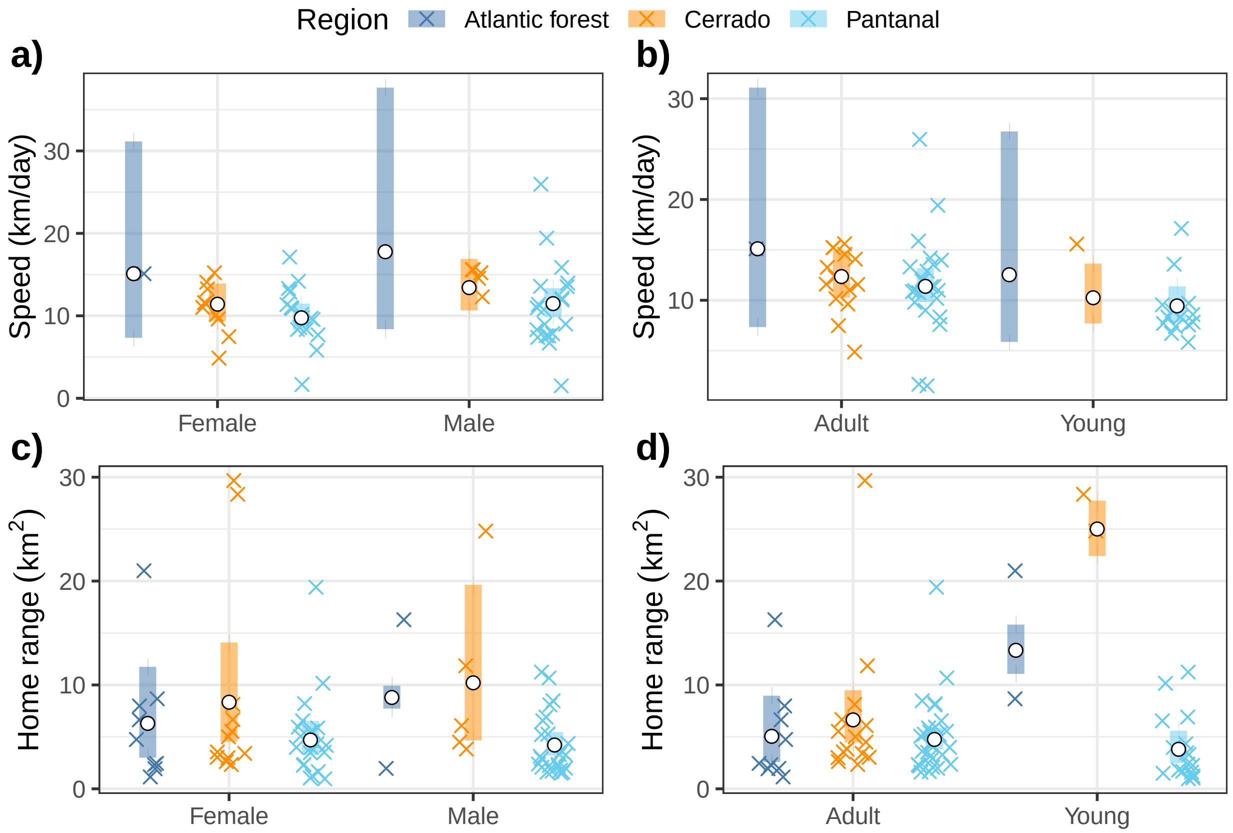

Region
Atlantic forest
Cerrado
Pantanal
a)
Speed (km/day)
0
10
20
30
Female
Male
b)
Speed (km/day)
10
20
30
Adult
Young
c)
Home range (km^2)
0
10
20
30
Female
Male
d)
Home range (km^2)
0
10
20
30
Adult
Young

3.5 Variation in movement across biomes and habitat composition (figure 5)

The best model is found using `MuMIn::dredge()`. Smooth terms are allowed to have a maximum `k` of 10 (or 5 if there are not enough unique values). The models are fit using maximum likelihood while using `MuMIn::dredge()`, but the best model is fit using Restricted Maximum Likelihood (REML).

```
tapirs.lu <- readRDS('../models/tapirs-land-use.rds') %>%
  mutate(tau.velocity.est = tau.velocity.est / (60^2), # from seconds to hours
         tau.velocity.low = tau.velocity.low / (60^2),
         tau.velocity.high = tau.velocity.high / (60^2))
tapirs.lu.sp <- filter(tapirs.lu, !is.na(speed.est))
tapirs.lu.tv <- filter(tapirs.lu, !is.na(tau.velocity.est))

# only use groups with at least 5 unique values
dplyr::select(tapirs.lu, `?`:plantation) %>%
  pivot_longer(-c()) %>%
  group_by(name) %>%
  summarize(unique = length(unique(value)),
            min = min(value),
            max = max(value)) %>%
  arrange(desc(unique)) %>%
  mutate(`> 5 uniques` = unique > 5,
         `> 10 uniques` = unique > 10)
```

```
## # A tibble: 11 x 6
##    name         unique   min    max `> 5 uniques` `> 10 uniques`
##    <chr>         <int> <dbl>  <dbl> <lgl>         <lgl>
##  1 forest           69 0.139 1      TRUE          TRUE
##  2 floodplain       62 0     0.656  TRUE          TRUE
##  3 savannah         18 0     0.611  TRUE          TRUE
##  4 dirt             12 0     0.221  TRUE          TRUE
##  5 pasture          11 0     0.352  TRUE          TRUE
##  6 crop              7 0     0.412  TRUE          FALSE
##  7 water             7 0     0.0942 TRUE          FALSE
##  8 ?                 6 0     0.181  TRUE          FALSE
##  9 headquarters      6 0     0.0178 TRUE          FALSE
## 10 plantation        3 0     0.324  FALSE         FALSE
## 11 urban             2 0     0.0210 FALSE         FALSE
```

```
# Gamma GAM regression on home range estimate
dredge(global.model = gam(area.est ~
                            s(forest, k = 10) +
                            s(floodplain, k = 10) +
                            s(savannah, k = 10) +
                            s(dirt, k = 10) +
                            s(pasture, k = 5) +
                            s(crop, k = 5) +
                            s(water, k = 5),
                          family = Gamma('log'),
                          data = tapirs.lu,
                          na.action = na.fail,
                          method = 'ML'), # need to use ML with dredge()
       rank = 'AICc') %>%
  head(20)
```

```
## Fixed term is "(Intercept)"
```

```
## Global model call: gam(formula = area.est ~ s(forest, k = 10) + s(floodplain, k = 10) + 
##     s(savannah, k = 10) + s(dirt, k = 10) + s(pasture, k = 5) + 
##     s(crop, k = 5) + s(water, k = 5), family = Gamma("log"), 
##     data = tapirs.lu, na.action = na.fail, method = "ML")
## ---
## Model selection table 
##     (Int) s(crp,5) s(drt,10) s(fld,10) s(frs,10) s(pst,5) s(svn,10) s(wtr,5) df   logLik  AICc delta weight
## 11  1.649                  +                   +                              8 -181.641 383.7  0.00  0.126
## 75  1.644                  +                   +                           +  9 -180.574 384.0  0.29  0.109
## 7   1.656                  +         +                                        8 -182.865 384.2  0.51  0.098
## 79  1.635                  +         +         +                           + 11 -178.892 384.9  1.17  0.070
## 15  1.642                  +         +         +                             10 -180.273 384.9  1.18  0.070
## 71  1.650                  +         +                                     +  9 -181.781 385.4  1.73  0.053
## 43  1.645                  +                   +                  +          10 -180.854 385.4  1.74  0.053
## 107 1.639                  +                   +                  +        + 11 -179.564 385.6  1.86  0.050
## 12  1.650        +         +                   +                              9 -181.662 385.6  1.92  0.048
## 76  1.643        +         +                   +                           + 10 -180.493 385.7  2.02  0.046
## 27  1.649                  +                   +        +                     9 -181.611 386.3  2.61  0.034
## 23  1.655                  +         +                  +                     9 -182.612 386.4  2.73  0.032
## 8   1.657        +         +         +                                        8 -182.964 386.5  2.78  0.031
## 80  1.635        +         +         +         +                           + 11 -178.829 386.6  2.87  0.030
## 91  1.644                  +                   +        +                  + 10 -180.526 386.6  2.94  0.029
## 16  1.642        +         +         +         +                             10 -180.298 386.7  3.05  0.027
## 39  1.658                  +         +                            +           8 -183.218 386.8  3.14  0.026
## 108 1.637        +         +                   +                  +        + 11 -179.276 387.1  3.39  0.023
## 3   1.685                  +                                                  5 -187.903 387.2  3.48  0.022
## 44  1.645        +         +                   +                  +          10 -180.835 387.3  3.65  0.020
## Models ranked by AICc(x)
```

Model 3 with `s(dirt)` as the only predictor is the best (parsimonious) model, since it is the best single-term model, and its AICc is not much larger than any of the models with a lower AICc (`delta` < 3.5). Still, the effect of `s(dirt)` is negligible.

```
m.hr <- gam(area.est ~ s(dirt, k = 5),
            family = Gamma('log'),
            data = tapirs.lu,
            method = 'REML')
summary(m.hr)
```

```
## 
## Family: Gamma 
## Link function: log 
## 
## Formula:
## area.est ~ s(dirt, k = 5)
## 
## Parametric coefficients:
##             Estimate Std. Error t value Pr(>|t|)    
## (Intercept)  1.68094    0.09319   18.04   <2e-16 ***
## ---
## Signif. codes:  0 '***' 0.001 '**' 0.01 '*' 0.05 '.' 0.1 ' ' 1
## 
## Approximate significance of smooth terms:
##           edf Ref.df     F p-value  
## s(dirt) 2.987  3.493 3.495  0.0228 *
## ---
## Signif. codes:  0 '***' 0.001 '**' 0.01 '*' 0.05 '.' 0.1 ' ' 1
```

```
##
## R-sq.(adj) =  0.477   Deviance explained = 28.6%
## -REML = 193.89  Scale est. = 0.64264   n = 74
```

```r
# Gamma GAM regression on average speed estimate
dredge(global.model = gam(speed.est ~
                            s(forest, k = 10) +
                            s(floodplain, k = 10) +
                            s(savannah, k = 10) +
                            s(dirt, k = 10) +
                            s(pasture, k = 5) +
                            s(crop, k = 5) +
                            s(water, k = 5),
                          family = Gamma('log'),
                          data = tapirs.lu.sp, # cannot have NAs
                          na.action = na.fail,
                          method = 'ML'),
       rank = 'AICc') %>%
  head(20)
```

```
## Fixed term is "(Intercept)"
```

```
## Global model call: gam(formula = speed.est ~ s(forest, k = 10) + s(floodplain, k = 10) +
##     s(savannah, k = 10) + s(dirt, k = 10) + s(pasture, k = 5) +
##     s(crop, k = 5) + s(water, k = 5), family = Gamma("log"),
##     data = tapirs.lu.sp, na.action = na.fail, method = "ML")
## ---
## Model selection table
##     (Int) s(crp,5) s(drt,10) s(fld,10) s(frs,10) s(pst,5) s(svn,10) s(wtr,5) df   logLik  AICc delta weight
## 73  2.400                                      +                           +  4 -148.964 306.8  0.00  0.226
## 105 2.399                                      +                  +        +  5 -148.673 308.6  1.86  0.089
## 77  2.399                            +         +                           +  5 -148.703 308.7  1.92  0.086
## 75  2.399                  +                   +                           +  5 -148.844 309.0  2.20  0.075
## 74  2.400        +                             +                           +  5 -148.919 309.1  2.35  0.070
## 89  2.400                                      +        +                  +  5 -148.946 309.2  2.41  0.068
## 97  2.405                                                         +        +  4 -150.688 310.2  3.45  0.040
## 9   2.409                                      +                              3 -152.014 310.5  3.76  0.035
## 65  2.409                                                                  +  3 -152.130 310.7  3.99  0.031
## 107 2.399                  +                   +                  +        +  6 -148.568 311.0  4.20  0.028
## 106 2.399        +                             +                  +        +  6 -148.604 311.0  4.27  0.027
## 101 2.402                            +                            +        +  5 -149.882 311.0  4.28  0.027
## 33  2.410                                                         +           3 -152.289 311.1  4.31  0.026
## 1   2.413                                                                     2 -153.438 311.1  4.35  0.026
## 109 2.399                            +         +                  +        +  6 -148.663 311.2  4.39  0.025
## 121 2.399                                      +        +         +        +  6 -148.672 311.2  4.41  0.025
## 93  2.399                            +         +        +                  +  6 -148.682 311.2  4.43  0.025
## 79  2.399                  +         +         +                           +  6 -148.683 311.2  4.43  0.025
## 78  2.399        +                   +         +                           +  6 -148.697 311.2  4.46  0.024
## 103 2.399                  +         +                            +        +  6 -148.733 311.3  4.53  0.023
## Models ranked by AICc(x)
```

No term contributed significantly to the model, since no model had an AICc lower than 4.35 with respect to the null model with only an intercept term (`speed.est ~ 1`, see model 1 in the table above).

```r
# Gamma GAM regression on average directional persistence (tau_v) estimate
filter(tapirs.lu, !is.na(tau.velocity.est)) %>%
  dplyr::select(`?`:plantation) %>%
  pivot_longer(-c()) %>%
  group_by(name) %>%
```

```r
  summarize(unique = length(unique(value)),
            min = min(value),
            max = max(value)) %>%
  arrange(desc(unique)) %>%
  mutate(`> 5 uniques` = unique > 5,
         `> 10 uniques` = unique > 10)
```

```
## # A tibble: 11 x 6
##    name         unique   min    max `> 5 uniques` `> 10 uniques`
##    <chr>         <int> <dbl>  <dbl> <lgl>         <lgl>
##  1 forest           46 0.229 1      TRUE          TRUE
##  2 floodplain       44 0     0.564  TRUE          TRUE
##  3 savannah         15 0     0.611  TRUE          TRUE
##  4 pasture          10 0     0.352  TRUE          FALSE
##  5 dirt              9 0     0.221  TRUE          FALSE
##  6 water             6 0     0.0942 TRUE          FALSE
##  7 crop              5 0     0.143  FALSE         FALSE
##  8 headquarters      5 0     0.0178 FALSE         FALSE
##  9 plantation        3 0     0.324  FALSE         FALSE
## 10 ?                 1 0     0      FALSE         FALSE
## 11 urban             1 0     0      FALSE         FALSE
```

```r
dredge(global.model = gam(tau.velocity.est ~
                            s(forest, k = 10) +
                            s(floodplain, k = 10) +
                            s(savannah, k = 10) +
                            s(dirt, k = 5) +
                            s(pasture, k = 5) +
                            s(crop, k = 5) +
                            s(water, k = 5),
                          family = Gamma('log'),
                          na.action = na.fail,
                          data = tapirs.lu.tv,
                          method = 'ML'),
       rank = 'AICc') %>%
  head(30)
```

```
## Fixed term is "(Intercept)"

## Global model call: gam(formula = tau.velocity.est ~ s(forest, k = 10) + s(floodplain,
##     k = 10) + s(savannah, k = 10) + s(dirt, k = 5) + s(pasture,
##     k = 5) + s(crop, k = 5) + s(water, k = 5), family = Gamma("log"),
##     data = tapirs.lu.tv, na.action = na.fail, method = "ML")
## ---
## Model selection table
##       (Int) s(crp,5) s(drt,5) s(fld,10) s(frs,10) s(pst,5) s(svn,10) s(wtr,5) df logLik  AICc delta weight
## 77  -0.8414                           +         +                            + 5 27.880 -44.3  0.00  0.138
## 105 -0.8409                                     +                  +         + 5 27.673 -43.8  0.41  0.112
## 73  -0.8370                                     +                            + 4 25.949 -42.9  1.34  0.071
## 79  -0.8421                  +        +         +                            + 6 28.200 -42.2  2.01  0.050
## 71  -0.8391                  +        +                                      + 5 26.845 -42.2  2.07  0.049
## 69  -0.8360                           +                                      + 4 25.520 -42.1  2.20  0.046
## 78  -0.8417        +                  +         +                            + 6 28.030 -41.9  2.35  0.043
## 109 -0.8415                           +         +                  +         + 6 27.939 -41.7  2.54  0.039
## 121 -0.8415                                     +        +         +         + 6 27.916 -41.7  2.58  0.038
## 103 -0.8415                  +        +                            +         + 6 27.913 -41.7  2.59  0.038
## 93  -0.8414                           +         +        +                   + 6 27.903 -41.7  2.61  0.037
```

```
## 101 -0.8381                   +                             +         +  5 26.426 -41.4  2.91  0.032
## 106 -0.8410       +                      +                  +         +  6 27.679 -41.2  3.06  0.030
## 107 -0.8409               +              +                  +         +  6 27.673 -41.2  3.07  0.030
## 70  -0.8378       +                +                                  +  5 26.284 -41.1  3.19  0.028
## 102 -0.8404       +                +                         +         +  6 27.440 -40.7  3.53  0.024
## 89  -0.8371                              +         +                  +  5 25.997 -40.5  3.77  0.021
## 75  -0.8370               +              +                            +  5 25.965 -40.4  3.83  0.020
## 74  -0.8370       +                      +                            +  5 25.949 -40.4  3.86  0.020
## 65  -0.8299                                                           +  3 23.118 -39.7  4.60  0.014
## 87  -0.8391               +        +               +                  +  6 26.868 -39.6  4.68  0.013
## 72  -0.8391       +       +        +                                  +  6 26.854 -39.6  4.71  0.013
## 85  -0.8360                        +               +                  +  5 25.521 -39.5  4.72  0.013
## 111 -0.8421               +        +     +                   +         +  7 28.214 -39.5  4.78  0.013
## 80  -0.8421       +       +        +     +                            +  7 28.207 -39.5  4.79  0.013
## 95  -0.8421               +        +     +         +                  +  7 28.204 -39.5  4.80  0.013
## 119 -0.8418               +        +               +         +         +  7 28.087 -39.2  5.03  0.011
## 94  -0.8418       +                +     +         +                  +  7 28.042 -39.1  5.12  0.011
## 110 -0.8417       +                +     +                   +         +  7 28.031 -39.1  5.15  0.011
## 125 -0.8417                        +     +         +         +         +  7 28.028 -39.1  5.15  0.011
## Models ranked by AICc(x)
```

Model 73 (`tau.velocity.est ~ s(forest, k = 10) + s(water, k = 5)`) is the best two-term model. While it is not substantially better than model 65 (`tau.velocity.est ~ s(water, k = 5)`), the `forest` smooth term was included because of the high range in proportion of forested habitat (0.138-1).

```
m.tauv <- gam(tau.velocity.est ~ s(forest, k = 10) + s(water, k = 5),
              family = Gamma('log'),
              data = tapirs.lu,
              method = 'REML')
summary(m.tauv)
```

```
##
## Family: Gamma
## Link function: log
##
## Formula:
## tau.velocity.est ~ s(forest, k = 10) + s(water, k = 5)
##
## Parametric coefficients:
##             Estimate Std. Error t value Pr(>|t|)
## (Intercept) -0.83701    0.07236  -11.57 8.67e-15 ***
## ---
## Signif. codes:  0 '***' 0.001 '**' 0.01 '*' 0.05 '.' 0.1 ' ' 1
##
## Approximate significance of smooth terms:
##           edf Ref.df     F p-value
## s(forest)   1      1 2.945  0.0933 .
## s(water)    1      1 5.431  0.0245 *
## ---
## Signif. codes:  0 '***' 0.001 '**' 0.01 '*' 0.05 '.' 0.1 ' ' 1
##
## R-sq.(adj) =  0.0231   Deviance explained = 19.4%
## -REML = -19.808  Scale est. = 0.24084   n = 46
```

Create predictions for each model and plot them:

```
# lu on hr (panel 5a)
pred.lu.hr <- tibble(dirt = seq(0, 0.22, length.out = 100))
```

```
pred.lu.hr <- bind_cols(pred.lu.hr,
                        predict(m.hr, newdata = pred.lu.hr, se.fit = TRUE)) %>%
  mutate(est = exp(fit),
         lwr = exp(fit - 1.96 * se.fit),
         upr = exp(fit + 1.96 * se.fit))

ggplot() +
  geom_ribbon(aes(dirt, ymin = lwr, ymax = upr), pred.lu.hr, alpha = 0.2) +
  geom_line(aes(dirt, est), pred.lu.hr) +
  geom_point(aes(dirt, area.est, color = region.lab), tapirs.lu, alpha = 0.9) +
  scale_color_manual('Region', values = pal) +
  labs(x = 'Proportion of exposed soil in the habitat',
       y = expression('Home Range Area'~(km^2))) +
  theme(legend.position = 'top',
        panel.grid = element_blank())
```

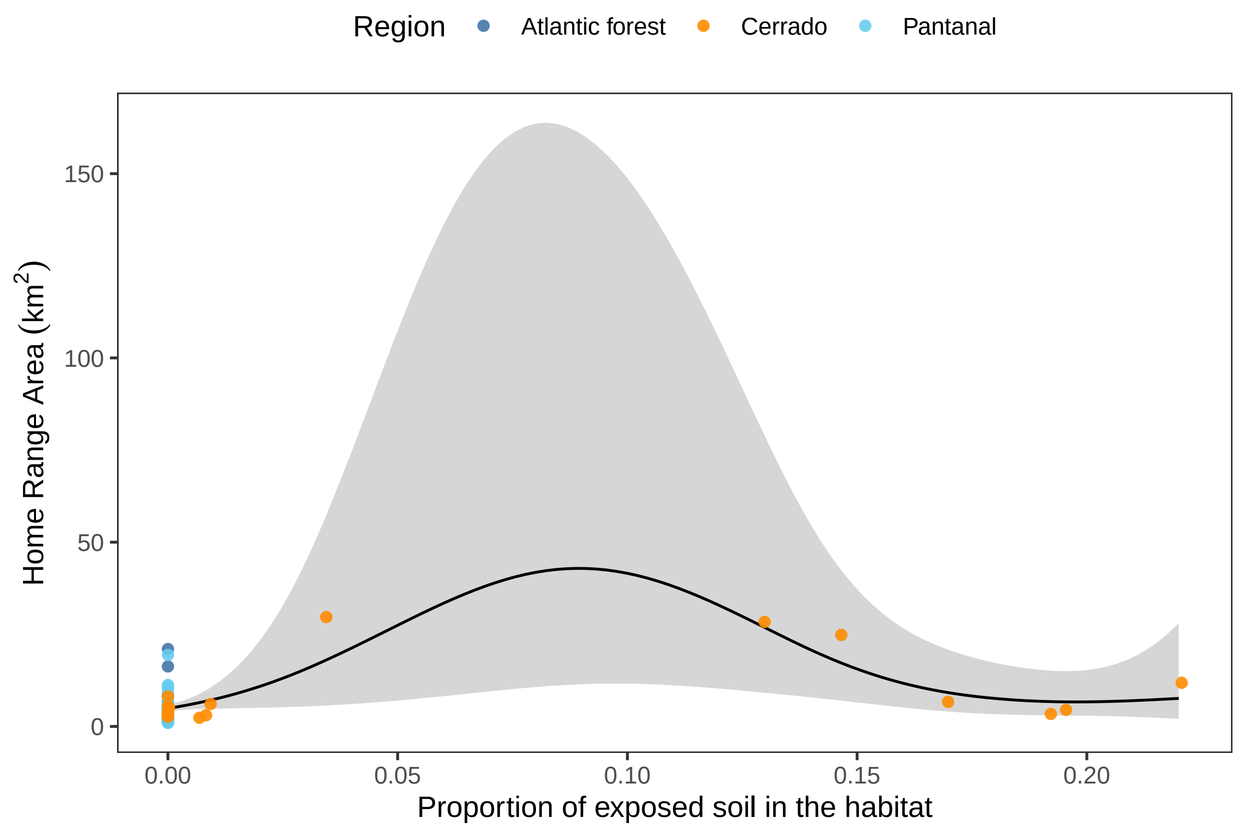


```
# forest on tau_v (5b)
newd.tv.f <- tibble(forest = seq_min_max(tapirs.lu$forest, n = 100),
                    water = mean(tapirs.lu$water, na.rm = TRUE))
pred.tv.f <-
  bind_cols(newd.tv.f, predict(m.tauv, newdata = newd.tv.f, se.fit = TRUE,
                               terms = 's(forest)')) %>%
  mutate(est = exp(fit),
         lwr = exp(fit - 1.96 * se.fit),
         upr = exp(fit + 1.96 * se.fit),
         model = 'Full dataset')
```

```r
ggplot() +
  geom_ribbon(aes(forest, ymin = lwr, ymax = upr), pred.tv.f, alpha = 0.2) +
  geom_line(aes(forest, est), pred.tv.f) +
  geom_segment(aes(x = forest, xend = forest, y = tau.velocity.low,
                   yend = tau.velocity.high, color = region.lab), tapirs.lu,
               lwd = 0.5, alpha = 0.5) +
  geom_point(aes(forest, tau.velocity.est, color = region.lab,
                 shape = tau.velocity.est > 1.5),
             filter(tapirs.lu, ! is.na(tau.velocity.est)), alpha = 0.9) +
  scale_shape_manual('Outlier', values = c(19, 4), labels = c('No', 'Yes')) +
  scale_color_manual('Region', values = pal[1:3]) +
  labs(x = 'Proportion of forested habitat',
       y = 'Directional persistence (h)') +
  theme(legend.position = 'top')
```

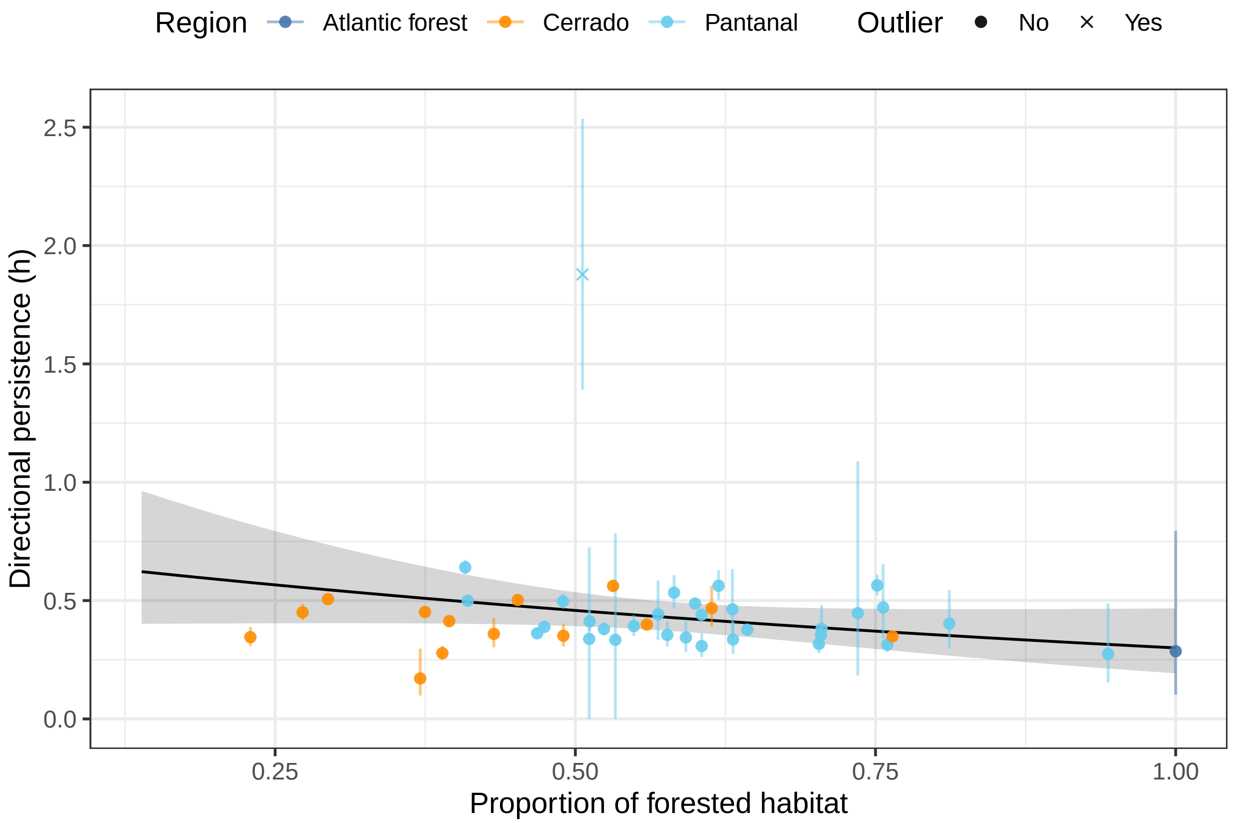

3.5.1 $\tau_v \sim$ s(forest) + s(water) without outlier

We can re-fit the best models for τ_v without the outlier:

```r
hist(tapirs.lu$tau.velocity.est, main = NULL, xlab = expression(widehat(tau[v])))
```

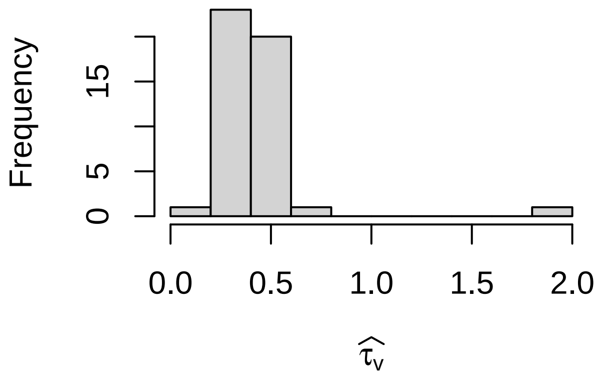


```
m.tauv.1 <- gam(tau.velocity.est ~ s(forest, k = 10) + s(water, k = 5),
                family = Gamma('log'),
                data = filter(tapirs.lu, tau.velocity.est < 1.5),
                method = 'REML')

# forest on tau_v (5b) without outlier
pred.tv.f <-
  bind_rows(pred.tv.f,
            bind_cols(newd.tv.f, predict(m.tauv.1, newdata = newd.tv.f, se.fit = TRUE,
                                         terms = 's(forest)')) %>%
              mutate(est = exp(fit),
                     lwr = exp(fit - 1.96 * se.fit),
                     upr = exp(fit + 1.96 * se.fit),
                     model = 'Without outlier'))
```

3.5.2 τ_v ~ s(forest) + s(water) without outlier or Atlantic forest data

We can repeat the analysis for panels 5b without the data from the Atlantic forest biome (after having removed the outlier):

```
m.tauv.2 <- gam(tau.velocity.est ~ s(forest, k = 10) + s(water, k = 5),
                family = Gamma('log'),
                data = filter(tapirs.lu,
                              tau.velocity.est < 1.5,
                              region != 'atlantica'),
                method = 'REML')

pred.tv.f <-
  bind_rows(pred.tv.f,
            bind_cols(newd.tv.f, predict(m.tauv.2, newdata = newd.tv.f, se.fit = TRUE,
                                         terms = 's(forest)')) %>%
              mutate(est = exp(fit),
                     lwr = exp(fit - 1.96 * se.fit),
                     upr = exp(fit + 1.96 * se.fit),
                     model = 'Without AF or outlier'))
```

```
ggplot() +
  geom_ribbon(aes(forest, ymin = lwr, ymax = upr, fill = model), pred.tv.f, alpha=0.2) +
  geom_line(aes(forest, est, color = model), pred.tv.f) +
  geom_errorbar(aes(x = forest, ymin = tau.velocity.low, ymax = tau.velocity.high,
                    color = region.lab), alpha = 0.5, tapirs.lu) +
  geom_point(aes(forest, tau.velocity.est, color = region.lab,
                 shape = tau.velocity.est > 1.5),
             filter(tapirs.lu, ! is.na(tau.velocity.est))) +
  scale_color_manual(NULL, values = c(pal[1:3], 1, 2, 'blue'),
                     aesthetics = c('color', 'fill'),
                     breaks = c(levels(tapirs.lu$region.lab), unique(pred.tv.f$model))) +
  scale_shape_manual('Outlier', values = c(19, 4), labels = c('No', 'Yes')) +
  labs(x = 'Proportion of forested habitat',
       y = 'Directional persistence (h)') +
  theme(legend.position = 'top')
```

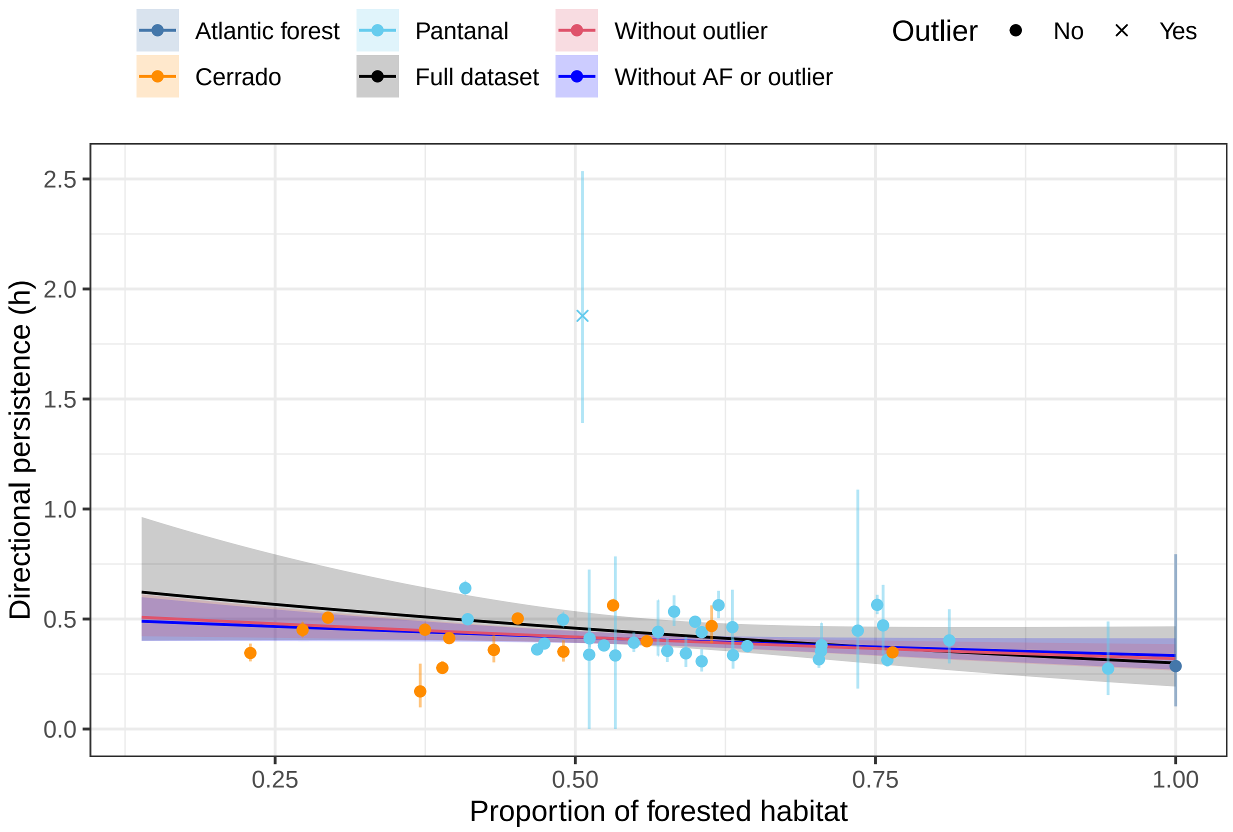

The removal of the outlier did not change the model's slope significantly, but it reduced model variance an appreciable amount. However, we do not have sufficient evidence to believe that the presence of the Atlantic forest data affected the model significantly, as its removal did not change the model slope substantially and the 95% confidence intervals of the two models (red and blue) mostly overlap.

3.6 Variation in movement across biomes and gradients of human disturbance (figure 6)

We can extract the ml-HFI for each tapir:

```
hfi.raster <- raster('../data/hfi-layers/ml_hfi_v1_2019.nc')

tapirs <-
  tapirs %>%
  mutate(region = factor(region), # need factors for GAMs
         hfi.mean = map_dbl(1:N, # add mean HFI
                            function(i)
                              extract(hfi.raster, as.sf(akde[[i]]))[[2]] %>% # take est
                              mean(na.rm = TRUE)),
         hr.size = map_dbl(akde, function(a) summary(a)$CI[2]))

# extract AKDE for the first tapir
joanna_akde <-
  SpatialPolygonsDataFrame.UD( # transform the AKDE to a spatial polygon
    object = tapirs$akde[[1]],
    proj4string = CRS(tapirs$data[[1]]@info$projection)) %>%
  spTransform(CRS("+proj=longlat")) %>% # change projection
  fortify() # change to data frame
```

```
## Regions defined for each Polygons
```

```
# import ml-HFI raster
joanna_raster <- hfi.raster %>%
  crop(c(xmin = -52.37, xmax = -52.33,
         ymin = -22.55, ymax = -22.50)) %>%
  rasterToPoints() %>%
  data.frame() %>%
  fortify() %>%
  rename(hfi = X__xarray_dataarray_variable__)

# create a plot of the cropped ml-HFI raster and the AKDE
ggplot() +
  geom_raster(aes(x, y, fill = hfi), joanna_raster) + # ml-HFI raster
  geom_polygon(aes(x = long, y = lat, lwd = group, lty = group), # AKDE
               joanna_akde, color = 'white', fill = '#80808040', show.legend = FALSE) +
  scale_fill_viridis_c('ml-HFI', limits = c(0, 1)) +
  scale_x_continuous('Longitude', expand = c(0, 0)) +
  scale_y_continuous('Latitude', expand = c(0, 0)) +
  scale_size_manual(breaks = unique(joanna_akde$group), values = c(.5, 1.25, .5)) +
  scale_linetype_manual(breaks = unique(joanna_akde$group), values = c(2, 1, 2)) +
  theme(panel.border = element_rect(colour = 'black', fill = 'transparent'),
        legend.position = 'top')
```

We can then estimate the effect of ml-HFI on tapir movement.

```
# hfi on hr size
m.hr.0 <- gam(hr.size ~ hfi.mean, # hfi.mean is linear on the link (log) scale
              family = Gamma('log'),
              data = tapirs,
              method = 'REML')
m.hr.1 <- gam(hr.size ~ s(hfi.mean), # allow hfi.mean to be smooth
```

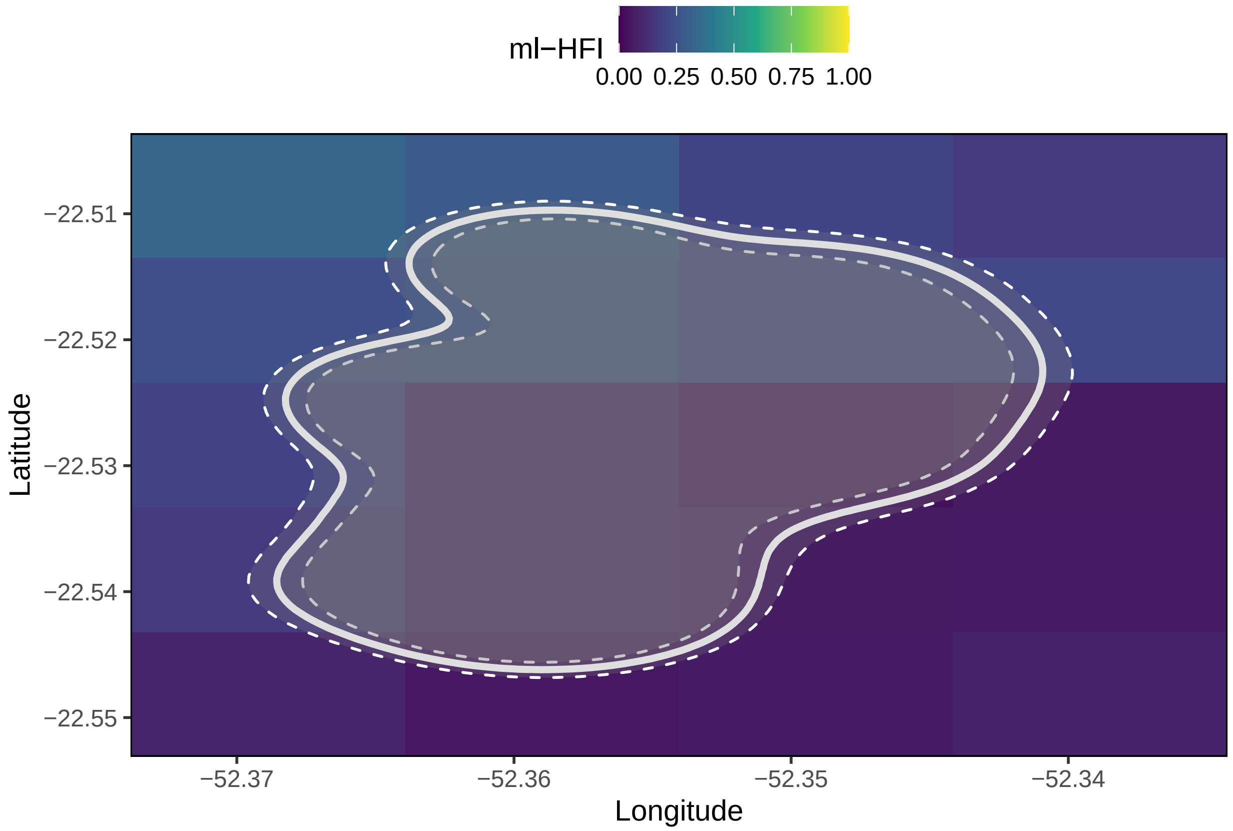

Figure 1: Autocorrelated Kernel Density Estimation for the home range of tapir labelled as "AF_01_JOANA". The solid line indicates the estimated 95% home range estimate, while the dashed lines indicate its 95% confidence inerval.

```r
              family = Gamma('log'),
              data = tapirs,
              method = 'REML')
m.hr.2 <- gam(hr.size ~ region + s(hfi.mean), # different intercept per region
              family = Gamma('log'),
              data = tapirs,
              method = 'REML')
m.hr.3 <- gam(hr.size ~ s(hfi.mean, by = region), # different smooth per region
              family = Gamma('log'),
              data = tapirs,
              method = 'REML')
m.hr.4 <- gam(hr.size ~ region + s(hfi.mean, by = region),
              family = Gamma('log'),
              data = tapirs,
              method = 'REML')

# accounting for effect of region does not improve the model fit
AIC(m.hr.0, m.hr.1, m.hr.2, m.hr.3, m.hr.4)
```

```
##                df      AIC
## m.hr.0 3.000000 444.6443
## m.hr.1 4.162897 444.5367
## m.hr.2 6.056456 447.4223
## m.hr.3 5.000777 444.0914
## m.hr.4 8.604185 447.7827
```

```r
summary(m.hr.0)
```

```
##
## Family: Gamma
## Link function: log
##
## Formula:
## hr.size ~ hfi.mean
##
## Parametric coefficients:
##              Estimate Std. Error t value Pr(>|t|)
## (Intercept)    2.0523     0.6760   3.036  0.00334 **
## hfi.mean      -0.3807     3.1431  -0.121  0.90393
## ---
## Signif. codes:  0 '***' 0.001 '**' 0.01 '*' 0.05 '.' 0.1 ' ' 1
##
##
## R-sq.(adj) =  -0.0138   Deviance explained = 0.0459%
## -REML = 219.04  Scale est. = 2.8666    n = 74
```

```r
# regression plot
pred.hr <- tibble(hfi.mean = seq(0.003, 0.31, length.out = 400))
pred.hr <- bind_cols(pred.hr,
                     predict(m.hr.0, newdata = pred.hr, se.fit = TRUE)) %>%
  mutate(est = exp(fit),
         lwr = exp(fit - 1.96 * se.fit),
         upr = exp(fit + 1.96 * se.fit))

ggplot() +
```

```r
  geom_ribbon(aes(hfi.mean, ymin = lwr, ymax = upr), pred.hr, alpha = 0.2) +
  geom_line(aes(hfi.mean, est), pred.hr) +
  geom_segment(aes(x = hfi.mean, xend = hfi.mean, y = area.low,
                   yend = area.high, color = region.lab), tapirs, lwd = 0.5,
               alpha = 0.5) +
  geom_point(aes(hfi.mean, hr.size, color = region.lab), tapirs, alpha = 0.9) +
  scale_color_manual('Region', values = pal[1:3]) +
  labs(x = 'ml-HFI', y = expression('Home Range Area'~(km^2))) +
  theme(legend.position = 'top')
```

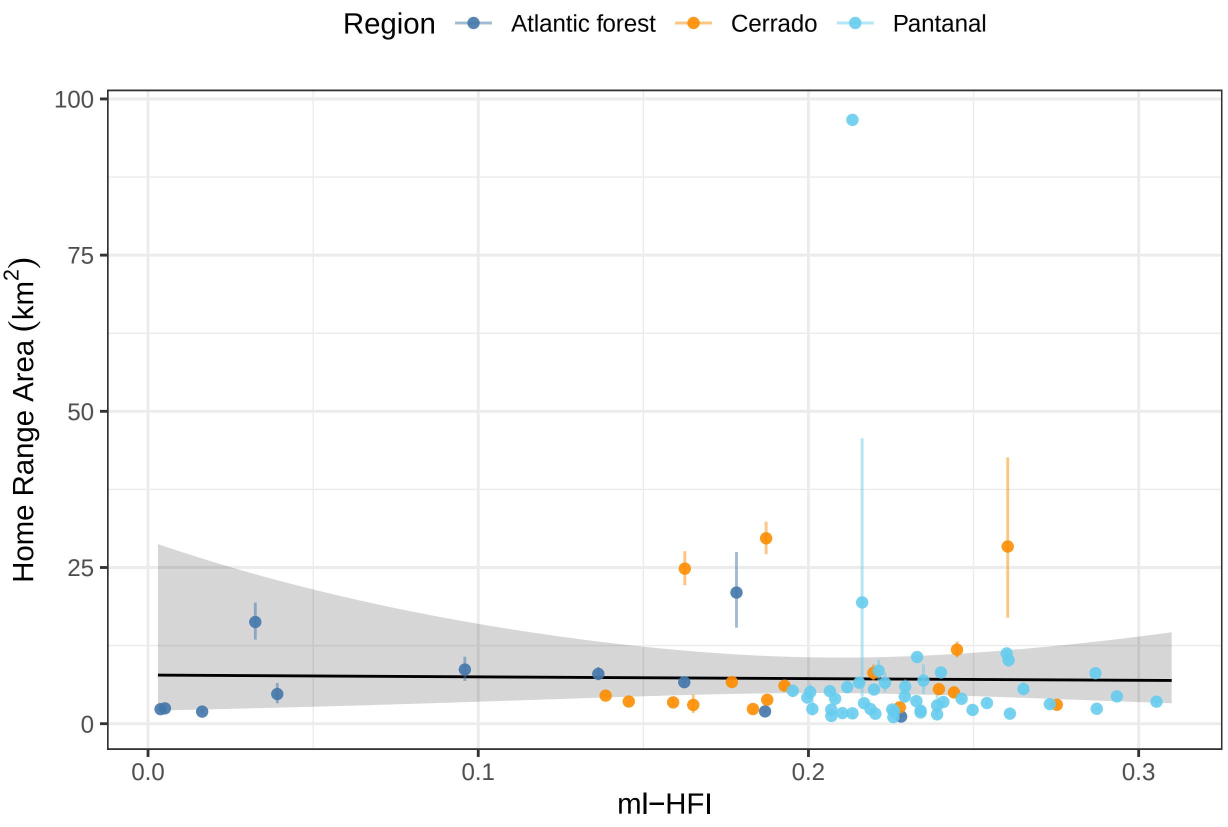


```r
# hfi on average speed
m.sp.0 <- gam(speed.est ~ s(hfi.mean), # shrunk a linear term by REML (edf == 1)
              family = Gamma('log'),
              data = tapirs,
              method = 'REML')
m.sp.1 <- gam(speed.est ~ region + s(hfi.mean),
              family = Gamma('log'),
              data = tapirs,
              method = 'REML')
m.sp.2 <- gam(speed.est ~ s(hfi.mean, by = region),
              family = Gamma('log'),
              data = tapirs,
              method = 'REML')
m.sp.3 <- gam(speed.est ~ region + s(hfi.mean, by = region),
              family = Gamma('log'),
              data = tapirs,
```

```r
              method = 'REML')

# accounting for effect of region does not improve the model fit
AIC(m.sp.0, m.sp.1, m.sp.2, m.sp.3)
```

```
##              df      AIC
## m.sp.0 3.000217 312.5687
## m.sp.1 5.000033 315.1423
## m.sp.2 5.001660 316.2714
## m.sp.3 6.000541 317.2902
```

```r
summary(m.sp.0)
```

```
##
## Family: Gamma
## Link function: log
##
## Formula:
## speed.est ~ s(hfi.mean)
##
## Parametric coefficients:
##             Estimate Std. Error t value Pr(>|t|)
## (Intercept)  2.41271    0.05133      47   <2e-16 ***
## ---
## Signif. codes:  0 '***' 0.001 '**' 0.01 '*' 0.05 '.' 0.1 ' ' 1
##
## Approximate significance of smooth terms:
##             edf Ref.df     F p-value
## s(hfi.mean)   1      1 0.397   0.532
##
## R-sq.(adj) =  -0.0099   Deviance explained = 0.631%
## -REML = 157.17  Scale est. = 0.13965   n = 53
```

```r
# regression plot
pred.sp <-
  bind_cols(dplyr::select(pred.hr, hfi.mean),
            predict(m.sp.0, newdata = pred.hr, se.fit = TRUE)) %>%
  mutate(est = exp(fit),
         lwr = exp(fit - 1.96 * se.fit),
         upr = exp(fit + 1.96 * se.fit))

p3 <-
  ggplot(tapirs) +
  geom_ribbon(aes(hfi.mean, ymin = lwr, ymax = upr), pred.sp, alpha = 0.2) +
  geom_line(aes(hfi.mean, est), pred.sp) +
  geom_segment(aes(x = hfi.mean, xend = hfi.mean, y = speed.low,
                   yend = speed.high, color = region.lab), lwd = 0.5,
               alpha = 0.5) +
  geom_point(aes(hfi.mean, speed.est, color = region.lab), alpha = 0.9)+
  scale_color_manual('Region', values = pal[1:3]) +
  labs(x = 'ml-HFI', y = 'Average speed (km/day)') +
  theme(legend.position = 'top'); p3
```

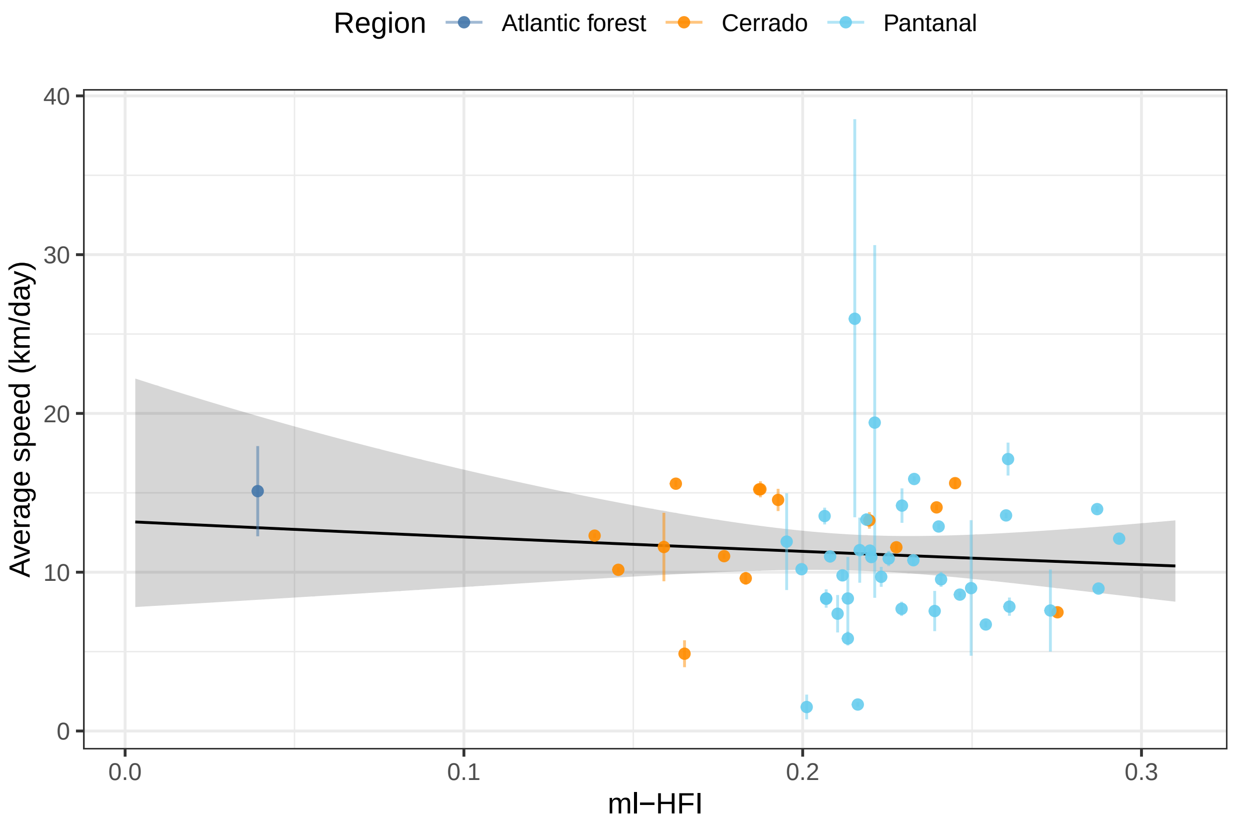

We can re-fit the regression model without the Atlantic forest data to show the data point (0.039, 15.1) has little effect on the conclusion drawn from the model (i.e. no significant effect of ml-HFI on average speed). The model without the Atlantic forest data point is indicated in red:

```
# without Mata Atlantica data
m.sp.0.a <- gam(speed.est ~ hfi.mean,
                family = Gamma('log'),
                data = filter(tapirs, region != 'atlantica'),
                method = 'REML')

pred.sp.a <-
  bind_cols(dplyr::select(pred.hr, hfi.mean),
            predict(m.sp.0.a, newdata = pred.hr, se.fit = TRUE)) %>%
  mutate(est = exp(fit),
         lwr = exp(fit - 1.96 * se.fit),
         upr = exp(fit + 1.96 * se.fit))

p3 +
  geom_ribbon(aes(hfi.mean, ymin = lwr, ymax = upr), pred.sp.a, alpha = 0.2,
              fill = 'red') +
  geom_line(aes(hfi.mean, est), pred.sp.a, color = 'red')
```

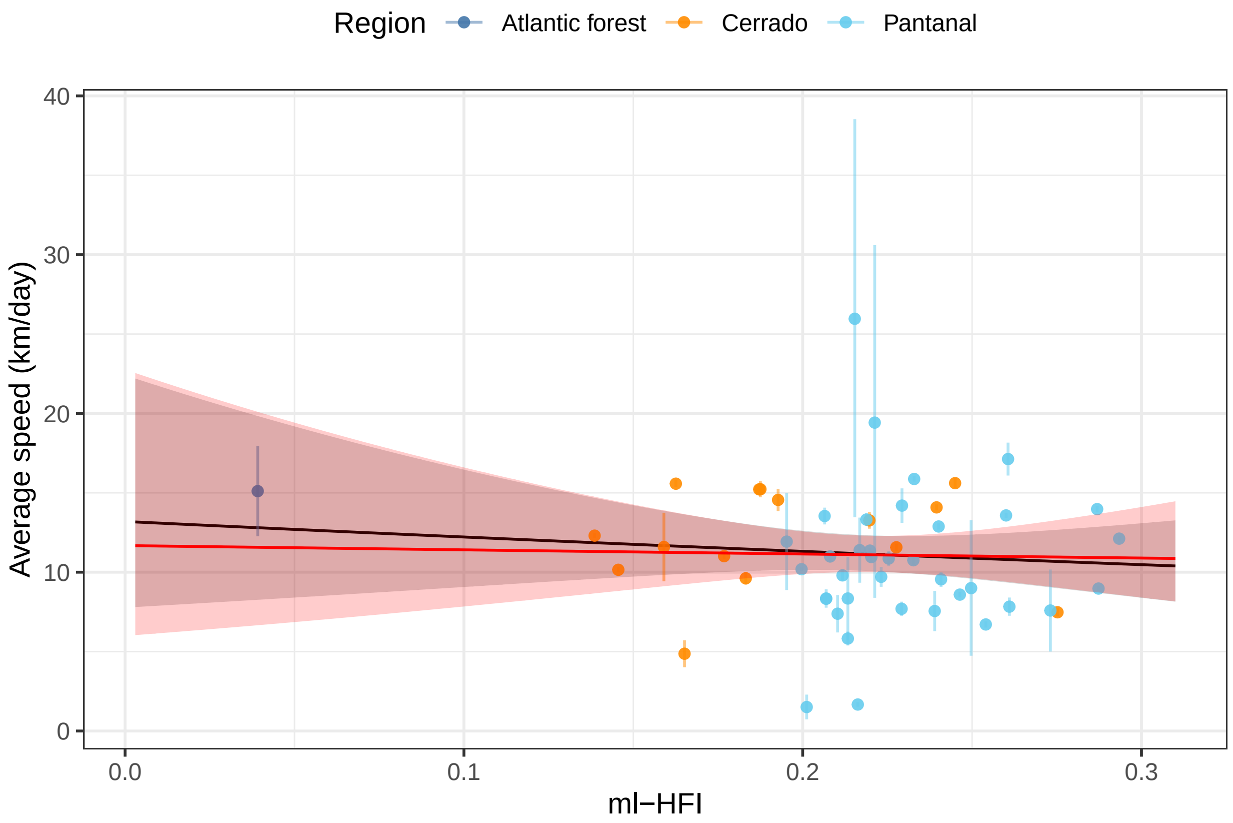

Region
Atlantic forest
Cerrado
Pantanal
40
30
20
10
0
Average speed (km/day)
0.0
0.1
0.2
0.3
ml−HFI

4 Core home range analysis

This section repeats the analyses from section 2 and 3 based on core (50%) home range estimates. All analyses which are not based on home range estimates (e.g., those on τ_p, τ_v) are not repeated.

4.1 Summary statistics for movement and space use parameters

```
# function to extract core home range
get_core_area <- function(i) {
  summary(tapirs$akde[[i]], units = FALSE, level.UD = 0.5)$CI %>%
    as.data.frame() %>%
    as_tibble()
}
```

```
tapirs <- readRDS('../models/tapirs-final.rds') %>% # tapir data
  arrange(name) %>%
  bind_cols(map_dfr(1:N, get_core_area) %>%
              transmute(core.area.low = low / 1e6,
                        core.area.est = est / 1e6,
                        core.area.high = high / 1e6)) %>%
  mutate(
    # translate name
    region.lab = if_else(region.lab == 'Mata Atlantica', 'Atlantic forest', region.lab)%>%
      factor())

tap <-
  tapirs %>%
  bind_rows(
    bind_cols(name = c('Atlantic forest', 'Cerrado', 'Pantanal', 'Overall'),
              region.lab = c('Atlantic forest', 'Cerrado', 'Pantanal', 'Overall'),
              # core home range estimates
              bind_rows(
                meta(tapirs$model[tapirs$region=='atlantica'], plot=F, level.UD=0.5)[1,],
                meta(tapirs$model[tapirs$region=='pantanal'], plot=F, level.UD=0.5)[1,],
                meta(tapirs$model[tapirs$region=='cerrado'], plot=F, level.UD=0.5)[1,],
                meta(tapirs$model, plot = FALSE, level.UD = 0.5)[1, ]) %>%
                rename(core.area.low = low, core.area.est = est, core.area.high=high)))%>%
  mutate(name = factor(name, levels = c(unique(tapirs$name), 'Atlantic forest',
                                        'Cerrado', 'Pantanal', 'Overall')),
         region.lab = factor(region.lab,
                             levels = c('Atlantic forest', 'Cerrado', 'Pantanal',
                                        'Overall')),
         average = if_else(name %in% c('Atlantic forest','Cerrado','Pantanal','Overall'),
                           'Group mean', 'Individual') %>%
           factor(levels = c('Individual', 'Group mean'))) %>%
  select(region.lab, name, core.area.low:core.area.high, average)
```

```
# est = estimated mean; low = lower 95% CI limit, high = upper 95% CI limit
tap %>%
  filter(average == 'Group mean') %>% # only group means
  select(-c(average, region.lab)) %>%
  relocate(name) %>% # move to be first column
  mutate(across(where(is.double), \(x) round(x, 2))) %>% # round to 2 decimals
  t() %>% # transpose to keep within page limits
```

```r
knitr::kable(caption = "Habitat-specific and overall means and 95% confidence intervals for the tapirs
```

Table 2: Habitat-specific and overall means and 95% confidence intervals for the tapirs' core home range estimates.

name	Atlantic forest	Cerrado	Pantanal	Overall
core.area.low	1.01	1.09	1.77	1.51
core.area.est	2.38	1.37	3.10	1.92
core.area.high	4.80	1.71	5.04	2.41

The overall mean core home range estimate was 1.92 km^2 with CI (1.51, 2.41) km^2 (see Table 2 above).

```r
range(tapirs$core.area.est) %>% round(digits = 1)
```

```
## [1] 0.2 6.8
```

The range in core home range area estimates was 0.2 - 6.8 km^2.

```r
# by sex
meta(list(female = filter(tapirs, sex == 'FEMALE')$akde,
          male = filter(tapirs, sex == 'MALE')$akde),
     plot = FALSE, verbose = TRUE, level.UD = 0.5) # verbose output with CIs
```

```
## * Sub-population female

##                         <U+0394>AICc
## inverse-Gaussian        0.000
## Dirac-d              6345.894

## * Sub-population male

##                         <U+0394>AICc
## inverse-Gaussian        0.000
## Dirac-d              7709.103

## * Joint population

##                         <U+0394>AICc
## inverse-Gaussian         0.00
## Dirac-d              14516.24

## * Joint population versus sub-populations (best models)

##                         <U+0394>AICc
## Joint population     0.000000
## Sub-population       3.875476

## $female
##                    low       est     high
## mean (km²)  0.9403064 1.2837582 1.709959
## CoV² (RVAR) 0.4875286 0.9179878 1.482389
## CoV  (RSTD) 0.7049614 0.9673503 1.229267
##
## $male
##                    low       est     high
## mean (km²)  0.8460187 1.1436247 1.512128
## CoV² (RVAR) 0.3546410 0.6993577 1.159155
## CoV  (RSTD) 0.6019908 0.8453665 1.088344
```

```
##
## $`mean ratio`
## , , low
##
##             /female     /male
## female/ 1.0000000 0.6996739
## male/   0.5549989 1.0000000
##
## , , est
##
##             /female    /male
## female/ 1.0000000 1.097596
## male/   0.8698682 1.000000
##
## , , high
##
##            /female    /male
## female/ 1.000000 1.641756
## male/   1.303228 1.000000
```

```
# by age group
meta(list(subadult = filter(tapirs, adult == 'No')$akde,
          adult = filter(tapirs, adult == 'Yes')$akde),
     plot = FALSE, verbose = TRUE, level.UD = 0.5)
```

```
## * Sub-population subadult

##                           &lt;U+0394&gt;AICc
## inverse-Gaussian        0.000
## Dirac-d              3254.431

## * Sub-population adult

##                           &lt;U+0394&gt;AICc
## inverse-Gaussian         0.00
## Dirac-d              11221.93

## * Joint population

##                           &lt;U+0394&gt;AICc
## inverse-Gaussian         0.00
## Dirac-d              14516.24

## * Joint population versus sub-populations (best models)

##                           &lt;U+0394&gt;AICc
## Sub-population       0.000000
## Joint population     1.125445

## $subadult
##                    low      est     high
## mean (km²)  0.7873994 1.406103 2.323720
## CoV² (RVAR) 0.5498715 1.589578 3.171346
## CoV  (RSTD) 0.7582676 1.289237 1.821015
##
## $adult
##                    low       est      high
## mean (km²)  0.9311746 1.1512015 1.4069680
## CoV² (RVAR) 0.3420499 0.5661251 0.8457361
```

```
## CoV  (RSTD) 0.5886384 0.7572862 0.9255956
##
## $`mean ratio`
## , , low
##
##           /subadult    /adult
## subadult/ 1.0000000 0.6117999
## adult/    0.3939304 1.0000000
##
## , , est
##
##           /subadult   /adult
## subadult/  1.000000 1.207801
## adult/     0.753792 1.000000
##
## , , high
##
##           /subadult   /adult
## subadult/  1.000000 2.035863
## adult/     1.398084 1.000000
```

Using `ctmm::meta()`, we found that core home range area was not significantly different ($\alpha = 0.05$) between sexes (females: mean = 1.28, 95% CI = (0.94, 1.71); males: mean = 1.14, 95% CI = (0.85, 1.51); LRT for female > male = 1.10, 95% CI = (0.70, 1.64)) nor between age groups (sub-adults: mean = 1.41, 95% CI = (0.79, 2.32); adults: mean = 1.15, 95% CI = (0.93, 1.41); LRT for sub-adult > adult = 1.21, 95% CI = (0.61, 2.04)).

4.2 Figures

4.2.1 AKDE 95% home range estimates (figure 2)

```
tapirs <- tapirs %>%
  mutate(proj = map(data, function(d) CRS(d@info$projection)),
         akde.df =
           map(1:N,
               function(i)
                 SpatialPolygonsDataFrame.UD(akde[[i]],
                                             proj4string = proj[[i]],
                                             level.UD = 0.5) %>% # core home range
                 spTransform(CRS("+proj=longlat")) %>%
                 fortify()))

atl.akdes <- bind_rows(tapirs$akde.df) %>% filter(grepl('AF_', group))
cer.akdes <- bind_rows(tapirs$akde.df) %>% filter(grepl('CE_', group))
pan.akdes <- bind_rows(tapirs$akde.df) %>% filter(grepl('PA_', group))

plot_grid(plot.akde('Atlantic Forest'), plot.akde('Cerrado'),
          plot.akde('Pantanal'), ncol = 2) # used ncol = 1 for figure in the main text
```

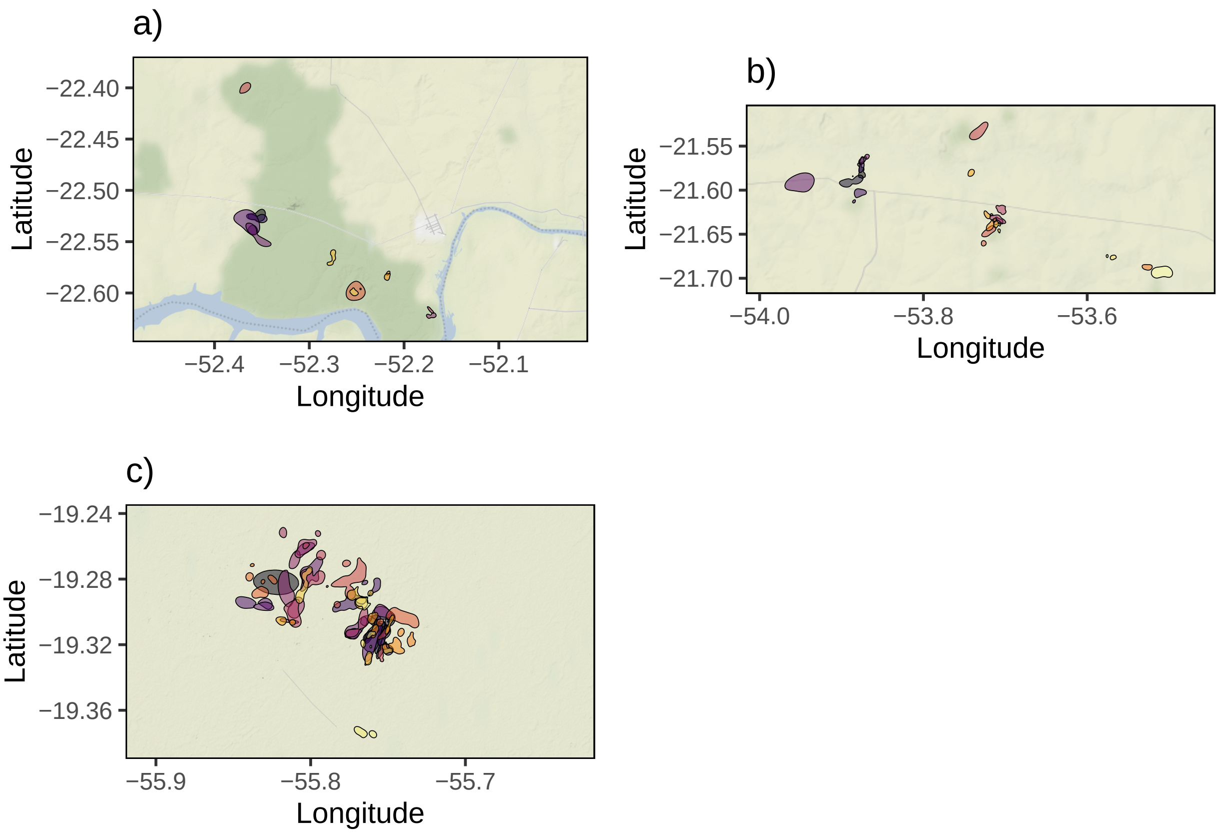

4.2.2 Parameter estimates using ctmm::meta() (figure 3)

```
# 3a) meta() of areas
ggplot(tap) +
  geom_segment(aes(x = core.area.low, xend = core.area.high, y = name, yend = name,
                   color = region.lab), lwd = 2) +
  geom_point(aes(x = core.area.est, y = name, shape = average), col = 'black', size=1.2) +
  geom_point(aes(x = core.area.est, y = name, shape = average), col = 'white', size=0.7) +
  scale_shape_manual(element_blank(), values = c(19, 17)) +
  scale_color_manual('Region', values = c(pal[1:3], 'black')) +
  scale_y_discrete(limits = rev,
                   labels = c('Means', 'Cerrado', 'Pantanal', 'Atlantic forest'),
                   breaks = c('Atlantic forest', 'CE_15_KURUKA',
                              'PA_33_GABRIELA', 'AF_14_JAMESBOND')) +
  labs(x = bquote('Estimated core home range area'~(km^2)),
       y = NULL)
```

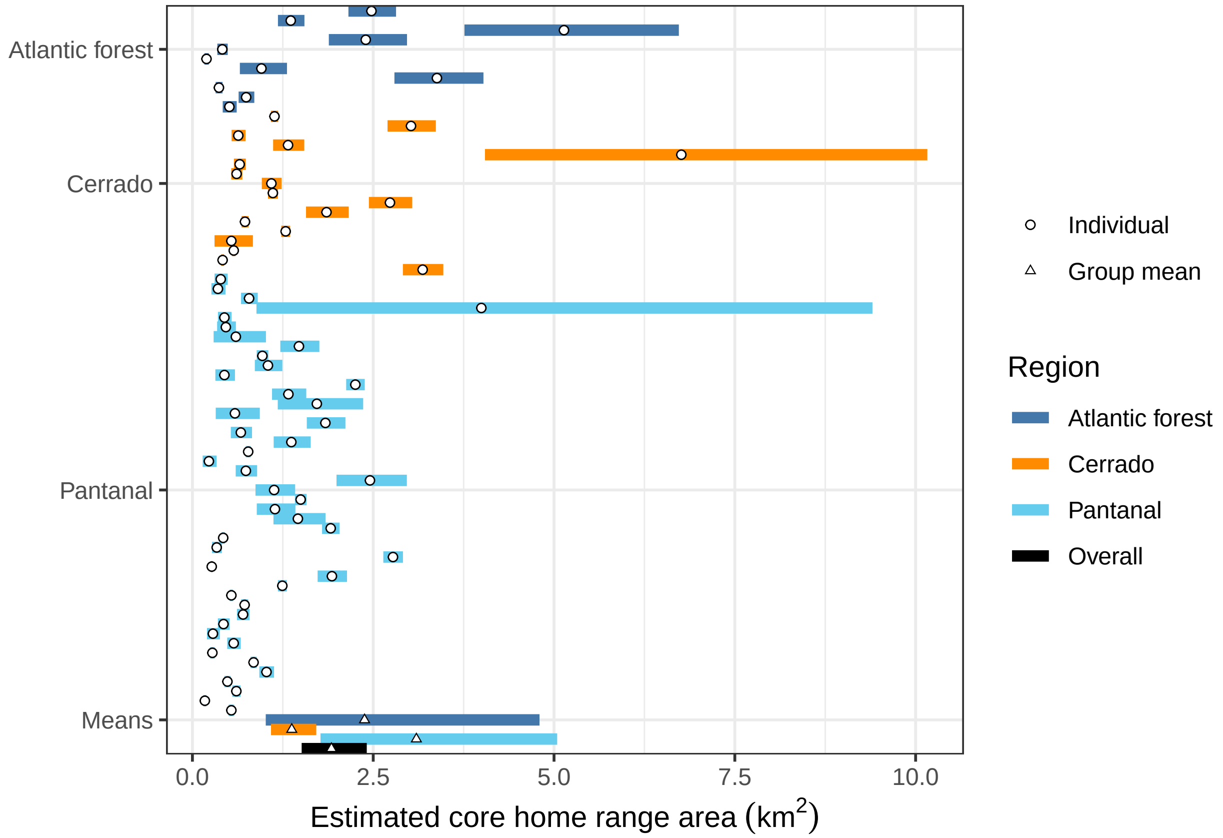

4.2.3 Strip charts (figure 4)

```
tapirs <- tapirs %>%
  mutate(sex = if_else(sex == 'FEMALE', 'Female', 'Male'),
         adult = if_else(adult == 'Yes', 'Adult', 'Young'),
         name = factor(name,
                       levels = c(unique(name), 'Atlantic forest', 'Cerrado', 'Pantanal',
                                  'Overall')),
         sex_r = paste(sex, region.lab, sep = '_') %>%
           factor(levels = c('Female_Atlantic forest', 'Female_Cerrado','Female_Pantanal',
                             'Male_Atlantic forest', 'Male_Cerrado', 'Male_Pantanal')),
         adult_r = paste(adult, region.lab, sep = '_') %>%
           factor(levels = c('Adult_Atlantic forest', 'Adult_Cerrado', 'Adult_Pantanal',
                             'Young_Atlantic forest', 'Young_Cerrado', 'Young_Pantanal')))

# removed analyses on speed

## core home range, sex
summ_cs <-
  expand_grid(sex = unique(tapirs$sex),
              region.lab = unique(tapirs$region.lab)) %>%
  mutate(bind_cols(map2_dfr(region.lab, sex,
                            \(x, y) hr_estimates(REGION = x, SEX = y, level.ud = 0.5))))

## core home range, adult
summ_ca <-
  expand_grid(adult = unique(tapirs$adult),
              region.lab = unique(tapirs$region.lab)) %>%
  mutate(bind_cols(map2_dfr(region.lab, adult,
                            \(x, y) hr_estimates(REGION = x, ADULT = y, level.ud = 0.5))))

# summary plots ----
hr_s <- summary_plot(group = 'sex', Y = 'core.area.est')
hr_a <- summary_plot(group = 'adult', Y = 'core.area.est')
```

```
plot_grid(get_legend(hr_s +
                       theme(legend.position = 'top')),
          plot_grid(hr_s, hr_a, ncol = 2,
                    labels = c('c)', 'd)')),
          ncol = 1, rel_heights = c(0.1, 1))
```

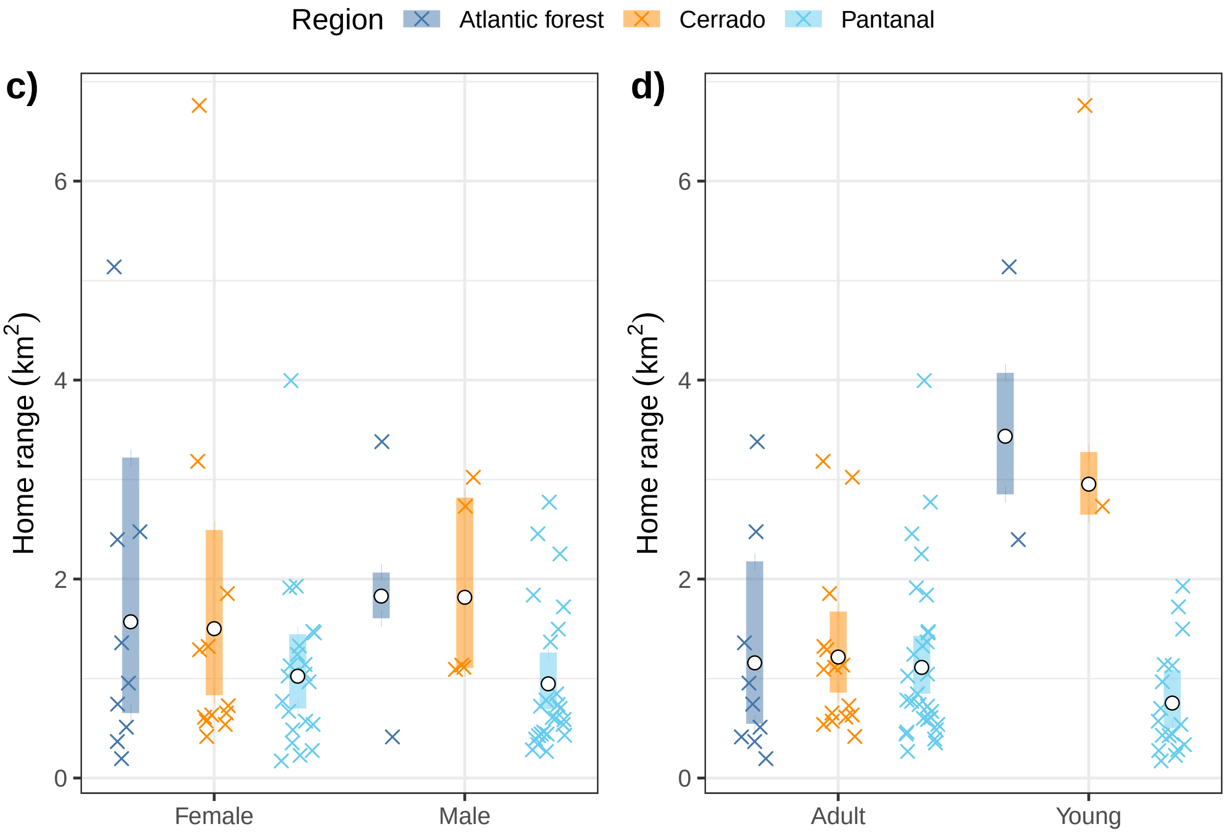

Region
Atlantic forest
Cerrado
Pantanal
c)
d)
Home range (km^2)
0
2
4
6
Female
Male
Adult
Young

4.2.4 Variation in movement across biomes and habitat composition (figure 5)

The best model is found using `MuMIn::dredge()`. Smooth terms are allowed to have a maximum `k` of 10 (or 5 if there are not enough unique values). The models are fit using maximum likelihood while using `MuMIn::dredge()`, but the best model is fit using Restricted Maximum Likelihood (REML).

```
tapirs.lu <- tapirs.lu %>%
  left_join(select(tapirs, name.short, core.area.est), by = 'name.short')
```

```
# Gamma GAM regression on home range estimate
dredge(global.model = gam(core.area.est ~
                            s(forest, k = 10) +
                            s(floodplain, k = 10) +
                            s(savannah, k = 10) +
                            s(dirt, k = 10) +
                            s(pasture, k = 5) +
                            s(crop, k = 5) +
                            s(water, k = 5),
                          family = Gamma('log'),
                          data = tapirs.lu,
                          na.action = na.fail,
                          method = 'ML'), # need to use ML with dredge()
       rank = 'AICc') %>%
  head(20)
```

```
## Fixed term is "(Intercept)"

## Global model call: gam(formula = core.area.est ~ s(forest, k = 10) + s(floodplain,
##     k = 10) + s(savannah, k = 10) + s(dirt, k = 10) + s(pasture,
##     k = 5) + s(crop, k = 5) + s(water, k = 5), family = Gamma("log"),
##     data = tapirs.lu, na.action = na.fail, method = "ML")
## ---
## Model selection table
##      (Int) s(crp,5) s(drt,10) s(fld,10) s(frs,10) s(pst,5) s(svn,10) s(wtr,5) df  logLik  AICc delta weight
## 73  0.1164                                      +                           +  7 -70.405 157.1  0.00  0.202
## 9   0.1255                                      +                              6 -71.960 158.4  1.28  0.107
## 74  0.1162        +                             +                           +  8 -70.359 158.9  1.86  0.080
## 89  0.1153                                      +        +                  +  8 -70.203 159.3  2.25  0.066
## 10  0.1238        +                             +                              7 -71.674 159.3  2.26  0.065
## 77  0.1162                            +         +                           +  8 -70.358 159.6  2.48  0.059
## 105 0.1165                                      +                  +        +  8 -70.409 159.6  2.52  0.057
## 75  0.1165                  +                   +                           +  8 -70.409 159.6  2.53  0.057
## 25  0.1242                                      +        +                     7 -71.734 160.6  3.56  0.034
## 11  0.1252                  +                   +                              7 -71.910 160.7  3.65  0.033
## 41  0.1255                                      +                  +           7 -71.948 160.8  3.68  0.032
## 13  0.1258                            +         +                              7 -71.999 160.8  3.74  0.031
## 90  0.1151        +                             +        +                  +  9 -70.164 161.3  4.18  0.025
## 76  0.1162        +         +                   +                           +  8 -70.355 161.3  4.26  0.024
## 78  0.1157        +                   +         +                           +  9 -70.280 161.4  4.31  0.023
## 70  0.1193        +                   +                                     +  8 -70.920 161.5  4.42  0.022
## 106 0.1162        +                             +                  +        +  8 -70.371 161.5  4.42  0.022
## 12  0.1239        +         +                   +                              8 -71.675 161.7  4.58  0.020
## 26  0.1217        +                             +        +                     8 -71.312 161.8  4.68  0.020
## 42  0.1238        +                             +                  +           8 -71.661 161.8  4.69  0.019
## Models ranked by AICc(x)
```

Model 9 with `s(forest)` as the only predictor is the best (parsimonious) model, since it is the best single-term model, and its AICc is not much larger than the model with the lowest AICc (`delta` = 1.28). Still, the effect of `s(forest)` is negligible.

```
m.chr <- gam(core.area.est ~ s(forest, k = 10), # core home range
             family = Gamma('log'),
             data = tapirs.lu,
             method = 'REML')
summary(m.chr)
```

```
##
## Family: Gamma
## Link function: log
##
## Formula:
## core.area.est ~ s(forest, k = 10)
##
## Parametric coefficients:
##             Estimate Std. Error t value Pr(>|t|)
## (Intercept)  0.12000    0.08413   1.426    0.158
##
## Approximate significance of smooth terms:
##             edf Ref.df     F p-value
## s(forest) 4.16  5.142 4.292 0.00179 **
## ---
## Signif. codes:  0 '***' 0.001 '**' 0.01 '*' 0.05 '.' 0.1 ' ' 1
##
## R-sq.(adj) =  0.297   Deviance explained = 29.2%
## -REML = 79.518  Scale est. = 0.52372   n = 74
```

```
# lu on hr (panel 5a)
pred.lu.hr <- tibble(forest = seq(0.13, 1, length.out = 300))
pred.lu.hr <- bind_cols(pred.lu.hr,
                        predict(m.chr, newdata = pred.lu.hr, se.fit = TRUE)) %>%
  mutate(est = exp(fit),
         lwr = exp(fit - 1.96 * se.fit),
         upr = exp(fit + 1.96 * se.fit))

ggplot() +
  geom_ribbon(aes(forest, ymin = lwr, ymax = upr), pred.lu.hr, alpha = 0.2) +
  geom_line(aes(forest, est), pred.lu.hr) +
  geom_point(aes(forest, core.area.est, color = region.lab), tapirs.lu, alpha = 0.9) +
  scale_color_manual('Region', values = pal[1:3],
                     labels = c('Atlantic forest', 'Cerrado', 'Pantanal')) +
  labs(x = 'Proportion of exposed soil in the habitat',
       y = expression('Home Range Area'~(km^2))) +
  theme(legend.position = 'top',
        panel.grid = element_blank())
```

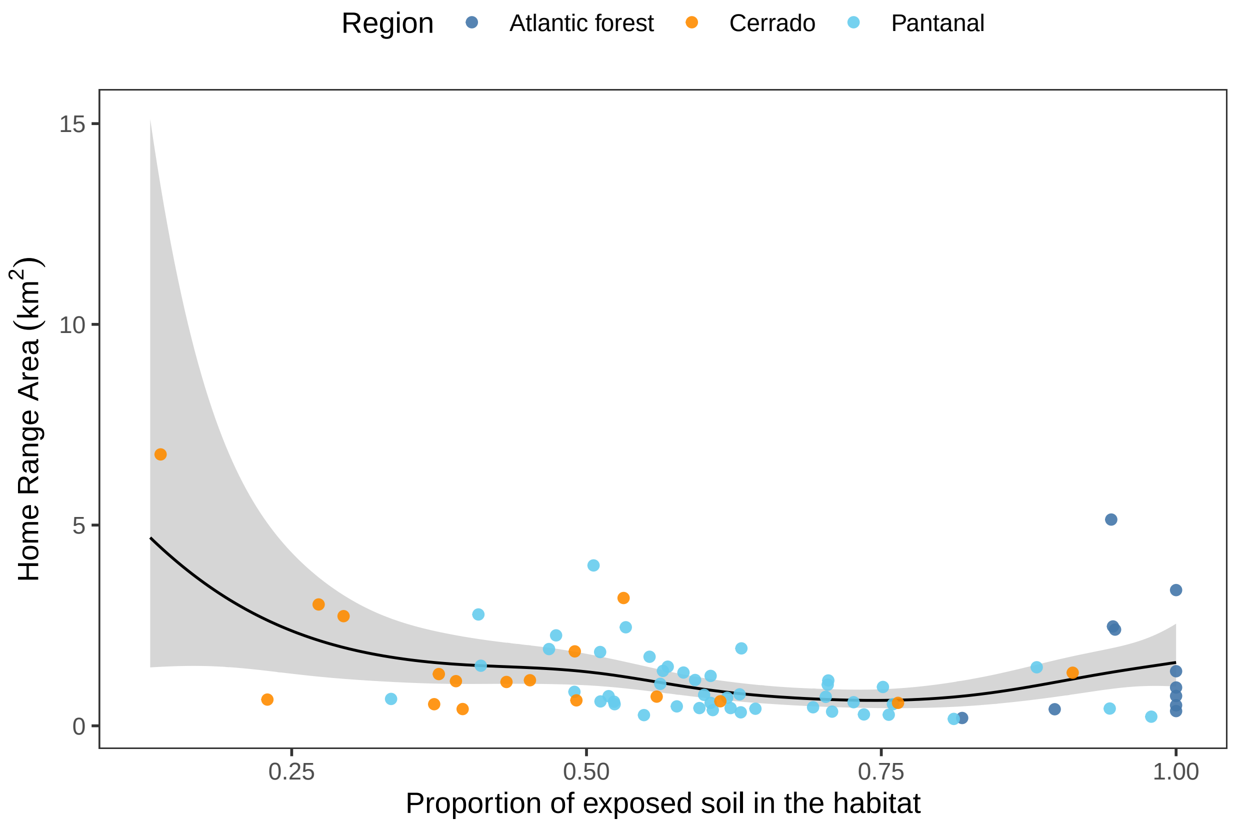
Region
Atlantic forest
Cerrado
Pantanal
Home Range Area (km^2)
15
10
5
0
0.25
0.50
0.75
1.00
Proportion of exposed soil in the habitat

4.2.5 Variation in movement across biomes and gradients of human disturbance (figure 6)

We can extract the ml-HFI for each tapir:

```
tapirs <-
  tapirs %>%
  mutate(region = factor(region), # need factors for GAMs
         hfi.mean.core = map_dbl(1:N, # add mean HFI
                                 function(i)
                                   extract(hfi.raster,
                                           as.sf(akde[[i]], level.UD = 0.5))[[2]] %>%
                                   mean(na.rm = TRUE)),
         core.hr.size = map_dbl(akde, function(a) summary(a, level.UD = 0.5)$CI[2]))

# extract AKDE for the first tapir
joanna_akde <-
  SpatialPolygonsDataFrame.UD( # transform the AKDE to a spatial polygon
    object = tapirs$akde[[1]],
    proj4string = CRS(tapirs$data[[1]]@info$projection),
    level.UD = 0.5) %>% # core home range
  spTransform(CRS("+proj=longlat")) %>% # change projection
  fortify() # change to data frame
```

```
## Regions defined for each Polygons
```

```
# import ml-HFI raster
joanna_raster <- hfi.raster %>%
  crop(c(xmin = -52.37, xmax = -52.33,
         ymin = -22.55, ymax = -22.50)) %>%
  rasterToPoints() %>%
  data.frame() %>%
  fortify() %>%
  rename(hfi = X__xarray_dataarray_variable__)

# create a plot of the cropped ml-HFI raster and the AKDE
ggplot() +
  geom_raster(aes(x, y, fill = hfi), joanna_raster) + # ml-HFI raster
  geom_polygon(aes(x = long, y = lat, lwd = group, lty = group), # AKDE
               joanna_akde, color = 'white', fill = '#80808040', show.legend = FALSE) +
  scale_fill_viridis_c('ml-HFI', limits = c(0, 1)) +
  scale_x_continuous('Longitude', expand = c(0, 0)) +
  scale_y_continuous('Latitude', expand = c(0, 0)) +
  scale_size_manual(breaks = unique(joanna_akde$group), # need 6 values because 2 areas
                    values = c(0.5, 0.5, 1.25, 1.25, 0.5, 0.5)) +
  scale_linetype_manual(breaks = unique(joanna_akde$group),
                        values = c(2, 2, 1, 1, 2, 2)) +
  theme(panel.border = element_rect(colour = 'black', fill = 'transparent'),
        legend.position = 'top')
```

We can then estimate the effect of ml-HFI on tapir movement.

```
# hfi on hr size
m.hr.0 <- gam(core.hr.size ~ hfi.mean.core, # hfi.mean is linear on the link (log) scale
              family = Gamma('log'),
              data = tapirs,
              method = 'REML')
```

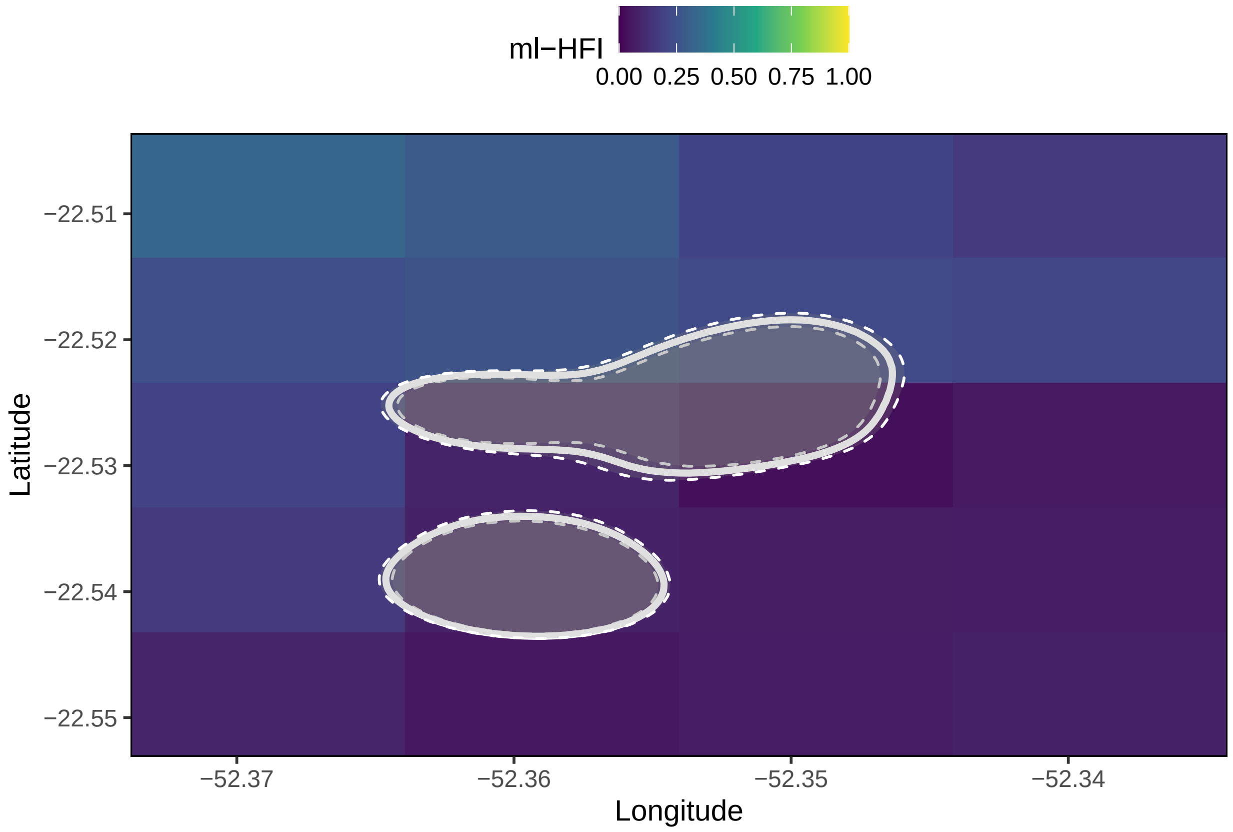

Figure 2: Autocorrelated Kernel Density Estimation for the home range of tapir labelled as "AF_01_JOANA". The solid line indicates the estimated 95% home range estimate, while the dashed lines indicate its 95% confidence inerval.

```r
m.hr.1 <- gam(core.hr.size ~ s(hfi.mean.core), # allow hfi.mean to be smooth
              family = Gamma('log'),
              data = tapirs,
              method = 'REML')
m.hr.2 <- gam(core.hr.size ~ region + s(hfi.mean.core), # different intercept per region
              family = Gamma('log'),
              data = tapirs,
              method = 'REML')
m.hr.3 <- gam(core.hr.size ~ s(hfi.mean.core, by = region), # different smooth per region
              family = Gamma('log'),
              data = tapirs,
              method = 'REML')
m.hr.4 <- gam(core.hr.size ~ region + s(hfi.mean.core, by = region),
              family = Gamma('log'),
              data = tapirs,
              method = 'REML')

# accounting for effect of region does not improve the model fit
AIC(m.hr.0, m.hr.1, m.hr.2, m.hr.3, m.hr.4)
```

```
##                df      AIC
## m.hr.0 3.000000 647.2564
## m.hr.1 4.424812 646.8630
## m.hr.2 6.284684 650.5878
## m.hr.3 5.823247 649.0566
## m.hr.4 8.537615 653.3486
```

```r
summary(m.hr.0)
```

```
##
## Family: Gamma
## Link function: log
##
## Formula:
## core.hr.size ~ hfi.mean.core
##
## Parametric coefficients:
##               Estimate Std. Error t value Pr(>|t|)
## (Intercept)     3.2948     0.3559   9.257 6.97e-14 ***
## hfi.mean.core   0.6355     1.6981   0.374    0.709
## ---
## Signif. codes:  0 '***' 0.001 '**' 0.01 '*' 0.05 '.' 0.1 ' ' 1
##
##
## R-sq.(adj) =  -0.00992   Deviance explained = 0.125%
## -REML = 320.07  Scale est. = 0.96612   n = 74
```

```r
# regression plot
pred.hr <- tibble(hfi.mean.core = seq(0.003, 0.31, length.out = 400))
pred.hr <- bind_cols(pred.hr,
                     predict(m.hr.0, newdata = pred.hr, se.fit = TRUE)) %>%
  mutate(est = exp(fit),
         lwr = exp(fit - 1.96 * se.fit),
         upr = exp(fit + 1.96 * se.fit))
```

```r
ggplot() +
  geom_ribbon(aes(hfi.mean.core, ymin = lwr, ymax = upr), pred.hr, alpha = 0.2) +
  geom_line(aes(hfi.mean.core, est), pred.hr) +
  geom_errorbar(aes(x = hfi.mean.core, ymin = core.area.low, ymax = core.area.high,
                    color = region.lab), tapirs, lwd = 0.5, alpha = 0.5) +
  geom_point(aes(hfi.mean.core, core.hr.size, color = region.lab), tapirs, alpha = 0.9) +
  scale_color_manual('Region', values = pal[1:3]) +
  labs(x = 'ml-HFI', y = expression('Core Home Range Area'~(km^2))) +
  theme(legend.position = 'top')
```

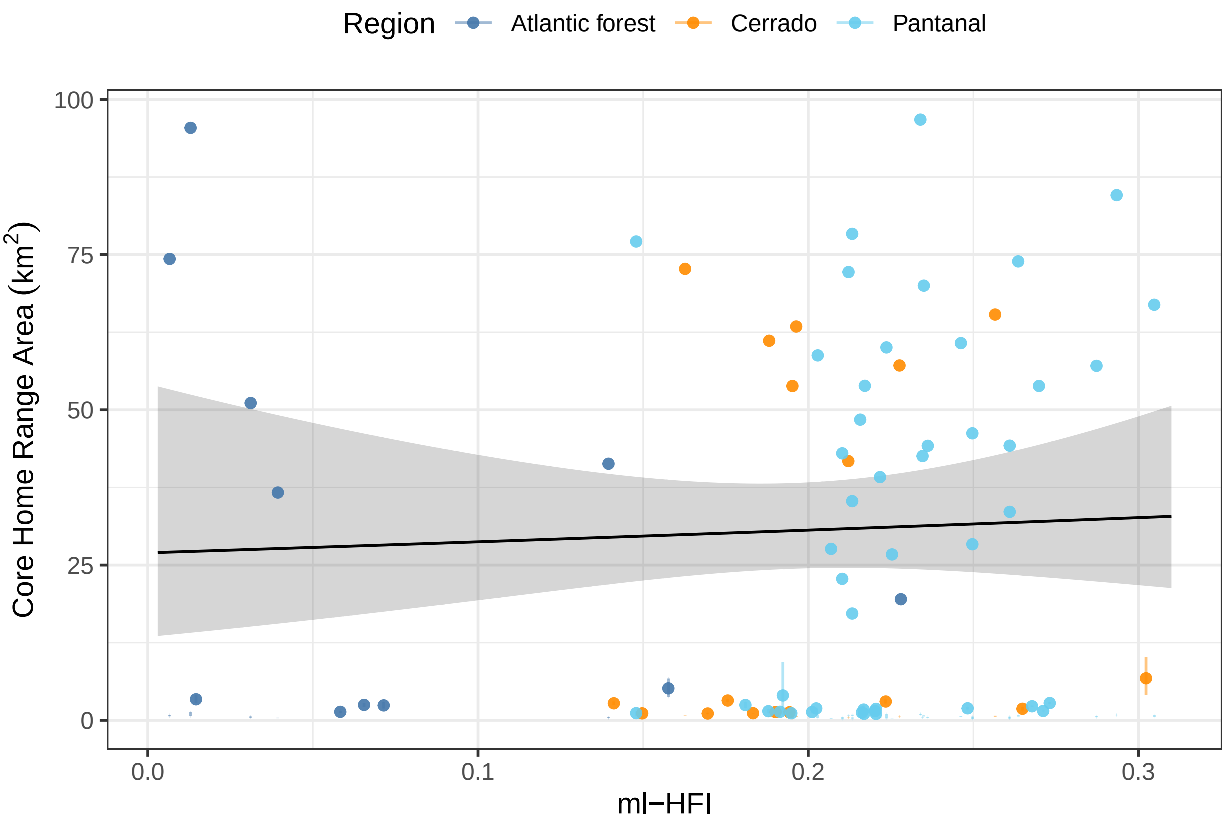

```r
sessionInfo()
```

```
## R version 4.1.2 (2021-11-01)
## Platform: x86_64-w64-mingw32/x64 (64-bit)
## Running under: Windows 10 x64 (build 19043)
##
## Matrix products: default
##
## locale:
## [1] LC_COLLATE=English_Canada.1252  LC_CTYPE=English_Canada.1252
## [3] LC_MONETARY=English_Canada.1252 LC_NUMERIC=C
## [5] LC_TIME=English_Canada.1252
##
## attached base packages:
## [1] stats     graphics  grDevices utils     datasets  methods   base
##
## other attached packages:
##  [1] ncdf4_1.18      dplyr_1.0.7     lubridate_1.8.0 gratia_0.6.9300
##  [5] cowplot_1.1.1   ggmap_3.0.0     ggplot2_3.3.5   raster_3.5-2
##  [9] sp_1.4-6        sf_1.0-4        MuMIn_1.43.17   tidyr_1.1.4
## [13] ctmm_0.6.1      mgcv_1.8-38     nlme_3.1-153    purrr_0.3.4
##
## loaded via a namespace (and not attached):
##  [1] httr_1.4.2          viridisLite_0.4.0   splines_4.1.2
##  [4] Bessel_0.6-0        assertthat_0.2.1    expm_0.999-6
##  [7] statmod_1.4.36      highr_0.9           stats4_4.1.2
## [10] yaml_2.2.1          numDeriv_2016.8-1.1 pillar_1.6.4
## [13] lattice_0.20-45     glue_1.5.1          digest_0.6.29
## [16] colorspace_2.0-2    htmltools_0.5.2     Matrix_1.3-4
## [19] plyr_1.8.6          pkgconfig_2.0.3     patchwork_1.1.1
## [22] scales_1.1.1        terra_1.4-20        jpeg_0.1-9
## [25] tibble_3.1.6        proxy_0.4-26        gmp_0.6-2.1
## [28] farver_2.1.0        generics_0.1.1      ellipsis_0.3.2
## [31] withr_2.4.3         cli_3.1.0           Rmpfr_0.8-7
## [34] magrittr_2.0.1      crayon_1.4.2        mvnfast_0.2.7
## [37] evaluate_0.14       fansi_0.5.0         class_7.3-19
## [40] tools_4.1.2         RgoogleMaps_1.4.5.3 lifecycle_1.0.1
## [43] stringr_1.4.0       munsell_0.5.0       compiler_4.1.2
## [46] e1071_1.7-9         rlang_0.4.12        classInt_0.4-3
## [49] units_0.7-2         grid_4.1.2          rstudioapi_0.13
## [52] rjson_0.2.20        labeling_0.4.2      bitops_1.0-7
## [55] rmarkdown_2.11      gtable_0.3.0        codetools_0.2-18
## [58] curl_4.3.2          DBI_1.1.1           R6_2.5.1
## [61] rgdal_1.5-27        knitr_1.36          rgeos_0.5-8
## [64] fastmap_1.1.0       utf8_1.2.2          KernSmooth_2.23-20
## [67] stringi_1.7.6       parallel_4.1.2      Rcpp_1.0.7
## [70] vctrs_0.3.8         png_0.1-7           tidyselect_1.1.1
## [73] xfun_0.28
```
